# Designing continuous equilibrium structures that counteract gravity in any orientation

**DOI:** 10.1038/s41598-023-34760-1

**Published:** 2023-05-17

**Authors:** Maria Redoutey, Evgueni T. Filipov

**Affiliations:** 1 Department of Civil and Environmental Engineering, University of Michigan, Ann Arbor, USA; 2 Department of Mechanical Engineering, University of Michigan, Ann Arbor, USA

**Keywords:** Engineering, Civil engineering

## Abstract

This paper presents a framework that can transform reconfigurable structures into systems with continuous equilibrium. The method involves adding optimized springs that counteract gravity to achieve a system with a nearly flat potential energy curve. The resulting structures can move or reconfigure effortlessly through their kinematic paths and remain stable in all configurations. Remarkably, our framework can design systems that maintain continuous equilibrium during reorientation, so that a system maintains a nearly flat potential energy curve even when it is rotated with respect to a global reference frame. This ability to reorient while maintaining continuous equilibrium greatly enhances the versatility of deployable and reconfigurable structures by ensuring they remain efficient and stable for use in different scenarios. We apply our framework to several planar four-bar linkages and explore how spring placement, spring types, and system kinematics affect the optimized potential energy curves. Next, we show the generality of our method with more complex linkage systems that carry external masses and with a three-dimensional origami-inspired deployable structure. Finally, we adopt a traditional structural engineering approach to give insight on practical issues related to the stiffness, reduced actuation forces, and locking of continuous equilibrium systems. Physical prototypes support the computational results and demonstrate the effectiveness of our method. The framework introduced in this work enables the stable, and efficient actuation of reconfigurable structures under gravity, regardless of their global orientation. These principles have the potential to revolutionize the design of robotic limbs, retractable roofs, furniture, consumer products, vehicle systems, and more.

## Introduction

Reconfigurable structures are systems with components that move, or *reconfigure*, along a prescribed kinematic path in order to achieve one or more functions. Examples include robots and robotic arms^1,2^, adaptive building facades^3^, deployable bridges and canopies^4,5^, stowable solar arrays^6^, furniture^7^, micro-grippers^8,9^, and retractable roofs^10^. For decades, research has focused on the kinematics, mobility, and stress states of reconfigurable structures^11–14^. A fundamental challenge that remains is efficiently actuating them while preserving stiffness and stability, especially in applications where gravity has a significant effect. In many designs, reconfiguration requires a large input of energy, resulting in inefficient, over-designed, and costly structures that are impractical to fabricate and operate.

Continuous equilibrium systems are a subset of reconfigurable structures with a kinematic mode that allows them to reconfigure with a negligible input of energy. Continuous equilibrium is also described as neutral stability or zero stiffness, and is characterized by a constant potential energy curve throughout reconfiguration^15–17^. Advantages of systems with continuous equilibrium include low energy required for actuation and an inherently stable reconfiguration path that avoids instabilities and dynamic snap-through behaviors.

Under gravity, most reconfigurable structures do not have continuous equilibrium with a constant potential energy curve; rather, the potential energy is affected by gravity as the structure moves through its kinematic path. The potential energy curve of a system is also affected by elements such as counterweights, springs, or magnets^18,19^. Continuous equilibrium is attained when the potential energy contributions of these components offset the potential energy due to gravity. Examples include the Anglepoise desk lamp, where pre-stressed springs allow the lamp to be easily repositioned^20^, bascule bridges which utilize a counterbalance to open^21,22^, and chairs which can be easily adjusted to recline at any angle^23^.

Continuous equilibrium has been attained in structures through the addition of zero-free-length springs^24,25^, an initial plastic deformation^26,27^, a temperature gradient^28^, thermal residual stresses^29^, or coupled components with offsetting deformations^30,31^. Structures can be designed to match a prescribed energy landscape (including a landscape corresponding to continuous equilibrium) by numerically computing the appropriate spring properties^32^. Despite these examples, there is currently no comprehensive framework to transform structures into systems with continuous equilibrium while considering gravity. In most previous studies, gravity has been ignored, or systems are either trivial to design or designed by trial and error. Additionally, all previous work has focused on achieving continuous equilibrium in only one specific orientation. If the entire structure is *reoriented* with respect to the ground (thus changing the potential energy curve due to gravity), continuous equilibrium is not maintained.

In this work, we present a framework to design structures that maintain continuous equilibrium as they *reconfigure* though their kinematic path and are *reoriented* with respect to a global reference frame. The method involves computing properties of springs that directly offset the potential energy due to gravity. In this paper, we first discuss the optimization setup used to find spring parameters that transform a simple linkage into a continuous equilibrium structure. Next, we explore how continuous equilibrium can be maintained as systems are reoriented. Then we apply the method to other linkages and expand to more complex systems. Finally, we use traditional structural engineering methods to explore the practical considerations for the use of these systems.

**Figure 1:**
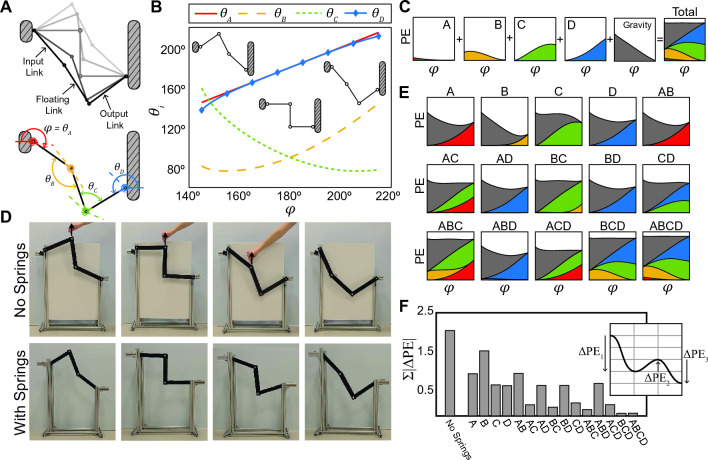
The Watt’s linkage. (**A**) The kinematics of the linkage are defined by the angle of the input link $\phi$. We define four locations for torsional springs, with angles $\theta _A, \theta _B, \theta _C,$ and $\theta _D$. (**B**) The angles of the four springs vary with $\phi$. (**C**) Potential energy contributions of four internal torsional springs (A, B, C, and D). When the spring contributions are summed with the contribution from gravity, the result is a nearly flat potential energy curve. (**D**) Experiments demonstrate how the Watt’s linkage with four internal torsional springs can be reconfigured to any position along its kinematic path, in contrast to the system with no springs, which collapses under gravity (see Movies S1 and S2). (**E**) Optimized potential energy curves for all possible combinations of internal torsional springs added to the Watt’s linkage. Placing springs only at locations B, C, and D is as effective as using all four springs. (**F**) Bar graph of the measure of the fluctuation in potential energy, $\Sigma | \Delta \text {PE}_{\text {T}}|$, for each spring combination case. The fluctuation of the illustrative curve shown in the inset equals $|\Delta \text {PE}_1| + |\Delta \text {PE}_2| + |\Delta \text {PE}_3|$.

## Potential energy of a four-bar linkage

We use planar four-bar linkages to demonstrate how simple reconfigurable structures can be transformed to have continuous equilibrium. Four-bar linkages are ubiquitous in engineering, found in robotics^33^, biomechanics and bio-inspired design^34–36^, automotive steering^37^, surgical instruments^38^, and many other fields. A planar four-bar linkage consists of four rigid members connected with pinned joints, resulting in a one-DOF mechanism^39^. The four bars are identified as the input link, output link, coupler (floating) link, and ground (fixed) link, which is an imaginary bar connecting the two support nodes (Fig. 1A). In this paper, we limit the kinematics of the linkages to a range where no link rotates a full $360^{\circ }$ with respect to an adjacent link. Although the kinematics^40–42^, dynamics^43,44^, design^45^, and inertia loads^46,47^ of four-bar linkages have been studied, the effect of gravity and self-weight on their mechanics is rarely considered. Static balancing of four-bar linkages involves counteracting gravity with springs, but previous approaches to static balancing are limited to specific linkages in a static orientation^38,48–50^. In contrast, our optimization approach can be applied to any arbitrary linkage at a range of orientations.

The four-bar linkage that we focus on in depth is the Watt’s linkage (Fig. 1A). The variation of the Watt’s linkage in this work consists of three bars of equal length (the imaginary fourth “bar,” or fixed link, connects the two support nodes). The left end of the input link is pinned one bar length above the right end of the output link. The kinematics of the Watt’s linkage are defined by the angle $\phi$, and we consider only a section of the kinematic path: $\phi _{min}=145^{\circ } \le \phi \le \phi _{max}=215^{\circ }$ (Fig. 1B). We focus on this range of $\phi$ because within this range, the midpoint of the floating link traces a nearly straight vertical path, a property which is exploited in applications such as vehicle suspension systems.

The potential energy of a bar *i* due to gravity is defined as $\text {PE}_{\text {G}i}(\phi ) = m_i * g * h_i(\phi ),$ where $m_i$ is the mass of bar *i*, $g = 9.81$ m/s$^2$, and $h_i$ is the height of the center of mass of bar *i*. The height is computed from a reference point 1 m below the support point of the output link. As the linkage moves through its kinematic path, the height of each bar changes, and so does the potential energy due to gravity; thus, $\text {PE}_{\text {G}i}$ is a function of $\phi$. We assume the bars of all linkages have a length of 0.3 m and a uniform mass distribution of 1 kg/m unless otherwise noted.

Our approach to achieving continuous equilibrium is to offset the potential energy due to gravity by adding springs, thus resulting in a flat total potential energy curve. We first add a torsional spring *j*, which has a linear stiffness $k_j$ (units: N-m/rad) and a rest angle $\alpha _j$ (units: rad). The potential energy in the spring is zero when the current angle of the spring $\theta _j$ is equal to the rest angle $\alpha _j$. The potential energy contribution of a torsional spring *j* is $\text {PE}_{\text {S}j}(\phi ) = \frac{1}{2} k_j (\theta _j(\phi ) - \alpha _j)^2$.

For a given configuration ($\phi$), the total potential energy of a system with *n* bars and *m* springs is expressed as

1 $$\begin{aligned} \text {PE}_{\text {T}}(\phi ) = \sum _{i}^{n} \text {PE}_{\text {G}i}(\phi ) + \sum _{j}^{m} \text {PE}_{\text {S}j}(\phi ). \end{aligned}$$

For an ideal system with continuous equilibrium, the $\text {PE}_{\text {T}}$ curve is perfectly flat. To quantify how flat the total potential energy curve is, we first compute the change in potential energy along the kinematic path, expressed as

2 $$\begin{aligned} \Delta \text {PE}_{\text {T}} = \frac{d \text { PE}_{\text {T}}(\phi )}{d \phi }. \end{aligned}$$

To compute the total change in potential energy, we integrate the absolute value of the difference along the kinematic path, expressed as

3 $$\begin{aligned} \Sigma \big |\Delta \text {PE}_{\text {T}}\big | = \int _{\phi _{min}}^{\phi _{max}} \Big |\frac{d \text { PE}_{\text {T}}(\phi )}{d \phi } \Big |d\phi . \end{aligned}$$

The quantity $\Sigma | \Delta \text {PE}_{\text {T}} |$ is a measure of the fluctuation in the $\text {PE}_{\text {T}}$ curve, where $\Sigma | \Delta \text {PE}_{\text {T}} |=0$ corresponds to a perfectly flat line.

## Results

### Optimizing spring properties for continuous equilibrium

We aim to minimize $\Sigma | \Delta \text {PE}_{\text {T}} |$ of a system by finding appropriate spring parameters (stiffnesses and rest angles) that result in springs that counteract the effect of gravity. To compute the spring properties, we minimize the $\Sigma | \Delta \text {PE}_{\text {T}} |$ using the MATLAB function **fmincon**. We define four possible locations for internal torsional springs on the Watt’s linkage, labelled A, B, C, and D in Fig. 1A. The design parameters for the optimization problem are the four spring stiffnesses ($k_A, k_B, k_C, k_D$) and four rest angles ($\alpha _A, \alpha _B, \alpha _C, \alpha _D$). The lower bound for the stiffness terms is 0 N-m/rad and the range for the rest angle $\alpha _j$ is limited to the kinematic path defined by the corresponding angle $\theta _j$ (Fig. 1B). There are no additional constraints placed on the optimization problem, which is expressed as

4 $$\begin{aligned} \min { }&\left( \sum \big |\Delta \text {PE}_{\text {T}} (\phi ) \big |\right) \\ \nonumber \quad \text {s.t.} \quad&k_j \ > \ 0\\ \nonumber&\alpha _j \ \epsilon \ [\theta _{j\text {min}}, \theta _{j\text {max}}]. \end{aligned}$$

The result of the optimization for the Watt’s linkage with internal torsional springs at all four locations is shown in Fig. 1C. The individual plots show the potential energy contributions of each spring and the total PE plot shows the aggregate result of all contributions, including gravity. The optimized spring parameters are $k_A = 0.396$ N-m/rad, $\alpha _A = 199^{\circ }$; $k_B = 1.18$ N-m/rad, $\alpha _B = 142^{\circ }$; $k_C = 1.23$ N-m/rad, $\alpha _C = 158^{\circ }$; and $k_D = 2.04$ N-m/rad, $\alpha _D = 139^{\circ }$. Qualitatively, the potential energy curve due to bar gravity is flattened with the addition of the potential energy stored in the springs. Quantitatively, we compare the optimized $\Sigma | \Delta \text {PE}_{\text {T}} |$ to the same measure considering only gravity, $\Sigma | \Delta \text {PE}_{\text {G}} |$. The $\Sigma | \Delta \text {PE}_{\text {G}} | = 2.07$ is reduced to $\Sigma | \Delta \text {PE}_{\text {T}} |= 0.065$ with the addition of springs; the $\text {PE}_{\text {T}}$ curve is 96.9% flatter than the $\text {PE}_{\text {G}}$ curve.

We compare all possible combinations of springs at locations A, B, C, and D that can be used in the optimization of the Watt’s linkage (Fig. 1E). Certain combinations are more effective than others at flattening the potential energy curve. For example, when optimizing the linkage with only a single spring, placing the spring at location D reduces $\Sigma | \Delta \text {PE}_{\text {T}} |$ more than placing it at locations A or B (Fig. 1F). As a result, when optimizing a linkage with more than one spring, the stiffness of springs A and B approach zero for combinations AD, BD, and ABD; the same $\Sigma | \Delta \text {PE}_{\text {T}} |$ can be achieved by placing a spring only at location D. Combination BCD offers effectively the same level of reduction as using all four springs. Results for all combinations are included in Table S1 of the Supporting Information.

Physical prototypes of the Watt’s linkage demonstrate how adding springs with optimized properties leads to a system with continuous equilibrium (Fig. 1D). The ideal properties of the springs were computed using the optimization framework, and springs with similar properties were used in the physical prototype (see Supporting Information and Table S14). Without springs, the Watt’s linkage collapses under gravity when a supporting force is removed (Movie S1). When the torsional springs are installed at locations A, B, C, and D, the linkage can be easily reconfigured into any position along its kinematic path (Movie S2). With springs, the linkage remains in the configuration in which it was placed and needs no additional forces to maintain its position. Notably, the properties of the store-bought springs do not perfectly match the calculated values; even with this deviation, the system with springs exhibits continuous equilibrium behavior. Using custom-made springs with properties that match the computed values would likely further improve the continuous equilibrium behavior exhibited by the physical structure.

### Reorientation of linkages

**Figure 2:**
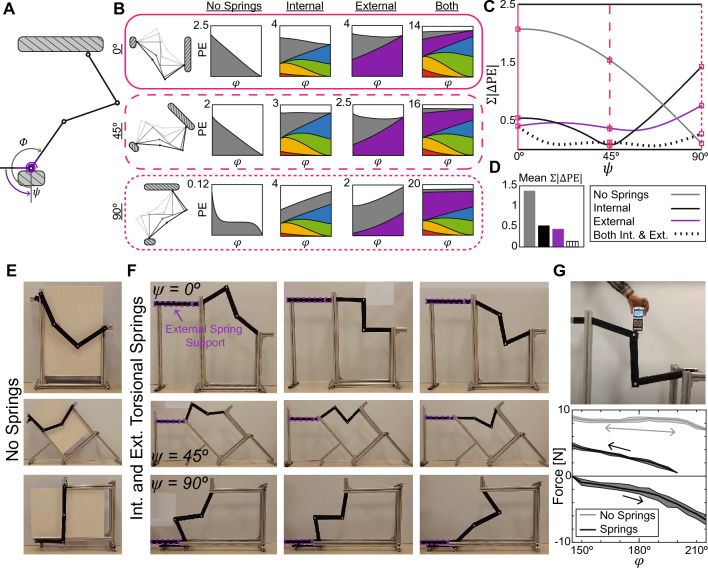
(**A**) The orientation of the Watt’s linkage is defined by $\psi$. An external torsional spring is connected to the input link and to a horizontal ground reference. (**B**) Potential energy curves for three orientations of the Watt’s linkage ($\psi = 0^{\circ }$, $\psi = 45^{\circ }$, $\psi = 90^{\circ }$). The linkage is optimized for cases with four internal torsional and/or one external torsional spring. (**C**) The measure of the fluctuation in the potential energy curve over the kinematic path with respect to orientation $\psi$. (**D**) When considering more than one orientation, spring parameters are found so that the mean($\Sigma | \Delta \text {PE}_{\text {T}}|$) is minimized. The case with both internal and external torsional springs results in the lowest mean($\Sigma | \Delta \text {PE}_{\text {T}}|$). (**E**) With no springs, the Watt’s linkage collapses due to gravity. (**F**) A prototype demonstrates that adding both internal and external torsional springs to the Watt’s linkage allows for continuous equilibrium reconfiguration at $\psi = 0^{\circ }$, $45^{\circ }$, and $90^{\circ }$. (**G**) The Watt’s linkage with springs requires a lower force for reconfiguration (measured using a force gauge as shown) than the system without springs.

In addition to reconfiguration through the kinematic path, structures can be *reoriented*, or rotated with respect to a global reference frame. For applications that require smooth motion in more than one orientation, such as robotics, it would be ideal to have one set of springs that ensure continuous equilibrium in all desired orientations. We define an orientation angle $\psi$ to describe the angle between a horizontal ground reference and the direction in which $\phi =0^{\circ }$ (Fig. 2(A)). To change orientation, the linkage is rotated about the support attached to the input link. In this paper, we consider a range of orientations $\psi = 0^{\circ }$ to $90^{\circ }$.

Figure 2B shows the potential energy curves and contributions for the Watt’s linkage at three orientations: $\psi = 0^{\circ }$, $45^{\circ }$, and $90^{\circ }$. The potential energy due to gravity (gray plots) is now dependent on the orientation of the linkage as well as the configuration (i.e., $\text {PE}_{\text {G}}(\phi ,\psi )$). For $\psi = 0^{\circ } \text { and } 45^{\circ }$, the system has a potential energy minimum at the end of the kinematic path; thus, the linkage collapses under gravity (Fig. 2E, Movie S1, Movie S3). For $\psi = 90^{\circ }$, the linkage has a region of constant potential energy in the middle of the kinematic path. However, if the linkage is pushed outside of this range, it also collapses (Movie S5).

To evaluate continuous equilibrium over different orientations, we plot the value of $\Sigma | \Delta \text {PE}_{\text {T}}|$ with respect to the orientation $\psi$ (Fig. 2C). Taking the mean of $\Sigma | \Delta \text {PE}_{\text {T}}|$ over the range of $\psi$ gives a measure of how close the structure is to continuous equilibrium at multiple orientations. As such, a mean($\Sigma | \Delta \text {PE}_{\text {T}}|$) = 0 indicates a structure with flat potential energy curves in *all* orientations. We use four internal torsional springs at locations A, B, C, and D to counteract gravity across multiple orientations. We set the optimization problem as:

5 $$\begin{aligned} \min { }&\left( \text {mean} \left( \sum \big |\Delta \text {PE}_{\text {T}} (\phi ,\psi ) \big | \right) \right) \\ \nonumber \quad \text {s.t.} \quad&k_j \ > \ 0 \\ \nonumber&\alpha _j \ \epsilon \ [\theta _{j\text {min}}, \theta _{j\text {max}}]. \end{aligned}$$

The potential energy in the internal springs does not change with respect to $\psi$, so their energy contributions are always the same, regardless of the orientation of the linkage (‘Internal’ column in Fig. 2B). As a result, $\Sigma | \Delta \text {PE}_{\text {T}} |$ is decreased more for some orientations than for others. The internal springs minimize $\Sigma | \Delta \text {PE}_{\text {T}} |$ most effectively for $\psi = 45^{\circ }$, where the resulting $\text {PE}_{\text {T}}$ curve is nearly flat (Fig. 2B-C). For $\psi = 90^{\circ }$, however, adding internal springs makes the potential energy curve less flat than it was initially ($\Sigma | \Delta \text {PE}_{\text {T}} |$ is increased compared to $\Sigma | \text {PE}_{\text {G}} |$). Because the objective is to minimize the mean($\Sigma | \Delta \text {PE}_{\text {T}} |$), the optimization does not necessarily lead to the smallest $\Sigma | \Delta \text {PE}_{\text {T}} |$ for each individual orientation. However, across the range of orientations, adding internal springs reduces the mean($\Sigma | \Delta \text {PE}_{\text {T}}|$) to 0.578 N-m from 1.382 N-m when no springs are used, a 58% decrease (Fig. 2C-D).

Because the potential energy due to gravity is dependent on $\psi$, we next consider adding a single external torsional spring with one end attached to the horizontal ground reference and one end attached to the input link of the Watt’s linkage (Fig. 2A). The potential energy of this external spring depends on both $\phi$ and $\psi$, because the rest angle $\alpha _{\text {E}}$ is defined with respect to the ground reference. The potential energy in the external spring is: $\text {PE}_{\text {E}} = \frac{1}{2}k_{\text {E}}(\phi - \alpha ^*)^2,$ where $\alpha ^* = \alpha _{\text {E}} + \psi$ accounts for the orientation of the linkage. The total potential energy for a system with an external torsional spring under gravity is expressed as

6 $$\begin{aligned} \text {PE}_{\text {T}}(\phi ,\psi ) = \sum _{i}^{n} \text {PE}_{\text {G}i}(\phi ,\psi ) + \text {PE}_{\text {E}}(\phi ,\psi ), \end{aligned}$$

and the optimization problem can be rewritten as

7 $$\begin{aligned} \min { }&\left( \text {mean} \left( \sum \big |\Delta \text {PE}_{\text {T}} (\phi ,\psi ) \big | \right) \right) \\ \nonumber \quad \text {s.t.} \quad&k_{\text {E}} \ > \ 0 \\ \nonumber&\alpha _{\text {E}} \ \epsilon \ [0, 2\pi ]. \end{aligned}$$

The Watt’s linkage optimized with one external torsional spring leads to a more effective minimization of the mean($\Sigma | \Delta \text {PE}_{\text {T}} |$) than the case with only internal torsional springs (Fig. 2B–D). For $\psi = 90^{\circ }$, $\Sigma | \Delta \text {PE}_{\text {T}} |$ is still higher than the case with no springs (Fig. 2C), but not as high as the internal spring case. The case with only one external spring reduces the mean($\Sigma | \Delta \text {PE}_{\text {T}} |$) by 67.8% to 0.445 N-m.

Finally, we consider adding both the four internal torsional springs and one external torsional spring. The total potential energy in the system for this case is expressed as

8 $$\begin{aligned} \text {PE}_{\text {T}}(\phi ,\psi ) = \sum _{i}^{n} \text {PE}_{\text {G}i}(\phi ,\psi ) + \sum _{j}^{m} \text {PE}_{\text {S}j}(\phi ) + \text {PE}_{\text {E}}(\phi ,\psi ). \end{aligned}$$

The design variables of the optimization problem are the stiffnesses and rest angles of all springs, internal and external, and the objective is again to minimize the mean($\Sigma | \Delta \text {PE}_{\text {T}}|$) over all desired orientations.

9 $$\begin{aligned} \min { }&\left( \text {mean} \left( \sum \big |\Delta \text {PE}_{\text {T}} (\phi ,\psi ) \big | \right) \right) \\ \nonumber \quad \text {s.t.} \quad&k_j \ > \ 0 \\ \nonumber&\alpha _j \ \epsilon \ [\theta _{j\text {min}}, \theta _{j\text {max}}]\\ \nonumber&k_{\text {E}} \ > \ 0 \\ \nonumber&\alpha _{\text {E}} \ \epsilon \ [0, 2\pi ] \end{aligned}$$

Optimizing both internal and external torsional springs significantly improves upon the results from the other two cases. The potential energy curves are nearly flat for $\psi = 0^{\circ }, 45^{\circ },$ and $90^{\circ }$ (Fig. 2B), and the $\Sigma | \Delta \text {PE}_{\text {T}} |$ is decreased for nearly all orientations (Fig. 2C). By adding both sets of torsional springs, we reduce mean($\Sigma | \Delta \text {PE}_{\text {T}} |$) to 0.137 N-m, a 90% reduction from the case with no springs (Fig. 2D). It is possible to reduce the mean($\Sigma | \Delta \text {PE}_{\text {T}} |$) further by limiting the system to a smaller range of $\psi$; for instance, the mean($\Sigma | \Delta \text {PE}_{\text {T}} |$) is 0.0602 N-m for a range of $\psi = 0^{\circ } \text { to } 30^{\circ }$. The optimized spring properties for all cases and several ranges of $\psi$ are included in Tables S2 and S3.

We fabricated a physical prototype of the Watt’s linkage with four internal torsional springs and one external torsional spring. Despite using springs with properties that deviate from the optimized solution (Tables S14 and S15), with both sets of torsional springs, the Watt’s linkage does not collapse and can be easily reconfigured at $\psi = 0^{\circ }$, $45^{\circ }$, and $90^{\circ }$ (Fig. 2F, Movie S4, Movie S6). We measure the vertical force required to reconfigure the Watt’s linkage using a force gauge (Fig. 2G). The linkage with springs requires a lower reconfiguration force than the system without springs; the absolute value of the force is reduced by 70% on average. Our testing set up allowed for either purely tensile (upward) or compressive (downward) testing, and thus we could not obtain a full range of data for the configurations of $\phi >200^{\circ }$. The direction of reconfiguration (up or down along the kinematic path) affects the magnitude of the force, which increases as the Watt’s linkage moves further from $\phi =180^{\circ }$, where the center point of the floating link no longer traces a vertical line. Friction in the system also contributes to the increase in force at the ends of the kinematic path; improved fabrication methods could minimize the effect of friction. Additionally, using springs with properties that better match the optimized solution could further reduce the force required for reconfiguration. The spring properties and details on the fabrication and testing are included in the Supporting Information.

### Effect of spring kinematic relationships on system performance

**Figure 3:**
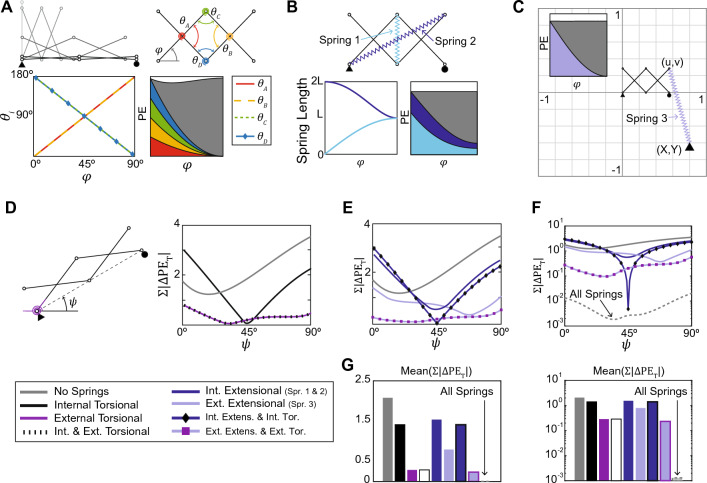
(**A**) The Scissor Mechanism has two sets of symmetric angles that are linearly related. (**B**) Adding two internal extensional springs or (**C**) one external extensional spring to the Scissor Mechanism results in total potential energy curves that are flatter than the case with four internal torsional springs. For the case with an external extensional spring, the anchor position (X, Y) and spring properties are found using optimization. (**D**) Reorientation of the Scissor Mechanism, considering several cases with torsional springs. The external spring reduces mean($\Sigma | \Delta \text {PE}_{\text {T}} |$) for the range of $\psi = 0^{\circ } \text { to } 90^{\circ }$. (**E**) Reorientation of the Scissor Mechanism for various cases with extensional springs. The case with one external extensional spring and one external torsional spring is the most effective at minimizing $\Sigma | \Delta \text {PE}_{\text {T}} |$ for nearly all orientations. (**F**) When all springs are added, $\Sigma | \Delta \text {PE}_{\text {T}} |$ is significantly reduced for all orientations. (G) Bar plots of the mean($\Sigma | \Delta \text {PE}_{\text {T}} |$) for all cases.

This section explores how system kinematics influence the performance of different spring types when optimizing for continuous equilibrium. We explore a Scissor Mechanism, where internal torsional springs can be placed in four locations, (A, B, C, and D in Fig. 3A). When optimized, the $\text {PE}_{\text {T}}$ curve is not as flat as the optimal result for the Watt’s linkage, and quantitatively $\Sigma | \Delta \text {PE}_{\text {T}} |$ is only reduced by 88% (from 1.77 N-m to 0.214 N-m). This smaller reduction is because the Scissor Mechanism is a symmetric linkage with all spring angles being linearly related: $\theta _A = \theta _B$, $\theta _C = \theta _D$, and $\theta _C = 180^{\circ } - \theta _A$ (Fig. 3A, Table S11). Thus, the potential energy due to the internal springs consists of four quadratic (2nd-order) terms; in fact, using a single or any combination of springs results in roughly the same overall performance (Figure S1 and Table S4). A contrasting example is a non-symmetric Double Rocker linkage, which has four angles with kinematic paths that are not symmetric nor linearly related, and include 1st, 3rd, and 4th order terms with respect to $\phi$ (Fig. S4, Table S13). The variety of higher order terms in $\text {PE}_{\text {T}}$ gives the system more freedom to offset the gravity curve and to reduce $\Sigma | \Delta \text {PE}_{\text {T}} |$ by more than 99% (Figure S3, Table S7).

From a practical perspective, the 88% improvement for the Scissor Mechanism may be sufficient to reduce actuation forces and improve stability. A physical prototype that would otherwise collapse under gravity (Movie S7) remains stable and requires a much lower reconfiguration force when four torsional springs are added (Fig. S6, Movie S8). For further improvement to the continuous equilibrium performance, we can also add *extensional springs*. The potential energy of an *internal extensional spring* *x* is $\text {PE}_{x} = \frac{1}{2}k_x\left( L_x-L_{0x}\right) ^2$, where *k* is the spring stiffness (units: N/m), $L_x(\phi )$ is the length of the spring which depends on the kinematics, and $L_0$ is the rest length (units: m). The extensional spring kinematics have sinusoidal relationships with respect to $\phi$ and are not symmetric with each other (Springs 1 and 2 in Fig. 3B, Table S12). This variation in kinematic relationships allows the extensional springs to minimize $\Sigma | \Delta \text {PE}_{\text {T}} |$ to 0.004 N-m (a 99.8% reduction). Another possibility is adding an *external extensional spring*, with one end attached to the Scissor Mechanism and the other end anchored to an external support (Spring 3, Fig. 3C). In this case, the design parameters of the optimization problem are the (X,Y) coordinates of the external anchor point along with the stiffness and rest length of the spring. The potential energy of the external extensional spring is $\text {PE}_X = \frac{1}{2}k_X\left( \sqrt{(u-X)^2+(v-Y)^2}-L_{0X}\right) ^2$, where $k_X$ is the spring stiffness (units: N/m), $L_{0X}$ is the rest length (units: m) and $(u(\phi ),v(\phi ))$ is the position of the point where the spring is attached to the Scissor Mechanism. Adding only this external extensional spring reduces $\Sigma | \Delta \text {PE}_{\text {T}} |$ from 1.77 to 0.0065 N-m (a 99.6% reduction). Another alternative that we have not explored here would be to use nonlinear springs^51^ and designing these springs to directly counteract the gravity curve.

We also consider the reorientation of the Scissor Mechanism from $\psi = 0^{\circ }$ to $90^{\circ }$ (Fig. 3D–G). Similar to the Watt’s linkage, adding an external torsional spring and attaching it to a horizontal ground reference is more effective at providing continuous equilibrium at different orientations because its potential energy is dependent on both configuration $\phi$ and orientation $\psi$ (Fig. 3D). The same is true when considering extensional springs, where a single spring attached to an external point provides a substantial advantage for obtaining continuous equilibrium in all orientations (Fig. 3E). Using all potential spring types in the optimization framework allows for near perfect continuous equilibrium performance in all orientations of the Scissor Mechanism (note the logarithmic scale in Fig. 3F). In reality, the case with only the external torsional and external extensional springs may suffice, as this combination provides a 89% reduction in the mean($\Sigma | \Delta \text {PE}_{\text {T}} |$). Results for all cases considered in these studies are included in the Supporting Information (Tables S5-S9, Figures S1-S4).

### Extension to various design cases

**Figure 4:**
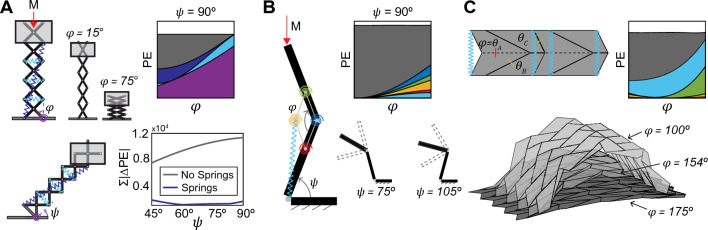
Examples of systems beyond simple linkages under gravity that can be designed to have continuous equilibrium: (**A**) A scissor lift counterbalances an external mass in any orientation between $\psi = 45^{\circ }$ and $90^{\circ }$, (**B**) a model of a knee exoskeleton supports a vertical load for different radial paths that change with orientation, and (**C**) a three-dimensional origami arch structure deploys from a flat state.

The optimization method can be expanded from simple four bar linkages to structures where additional complexity needs to be incorporated. We use the framework to design a scissor lift, a model of a knee, and an origami arch (Fig. 4). These examples add complexity by including an external mass carried along a linear or radial path and expanding the principles to a three-dimensional origami structure.

The scissor lift is a larger version of the Scissor Mechanism at $\psi = 90^{\circ }$, with equivalent kinematics and the addition of an external mass that is carried along a linear path. We model the linkage with all member lengths of 1 m, uniform mass distribution equal to 10 kg/m, and an external mass (to represent the weight of the basket and occupants) of M = 200 kg, with its center located at the midpoint of the last scissor unit (Fig. 4A). Based on the results in Fig. 3, we chose to use an external torsional spring and two internal extensional springs to obtain continuous equilibrium. At the orientation $\psi = 90^{\circ }$, adding these springs leads to a nearly flat potential energy curve where $\Sigma | \Delta \text {PE}_{\text {T}} |$ is reduced by 99.8%, from 9417.6 N-m to 16.95 N-m. This combination of springs reduces $\Sigma | \Delta \text {PE}_{\text {T}} |$ comparably for other orientations, and reduces the mean($\Sigma | \Delta \text {PE}_{\text {T}} |$) by 84% for a range of orientations $\psi = 45^{\circ }$ to $90^{\circ }$ (Figure S7).

Next, we model a knee exoskeleton as a planar linkage with two members resembling the human leg and four shorter bars of equal length positioned at the knee joint (Fig. 4B, Figure S8(A-B)). The structure carries an external mass along a variable radial path. The lower “calf” member defines the orientation $\psi$ of the system, while the upper “thigh” member reconfigures with kinematics defined by the angle $\phi$ with respect to the calf member. The self-weight of the members (2.5 kg each) is applied at their centroids and an external mass M = $30$ kg is applied at the top of the thigh member. After exploring different combinations, we chose to use four internal torsional springs and one internal extensional spring to obtain continuous equilibrium. The magnitude of the potential energy due to gravity of the system changes with the orientation $\psi$, but the *shape* of the $\text {PE}_{\text {G}}$ curve does not, so internal springs are sufficient to minimize the mean($\Sigma | \Delta \text {PE}_{\text {T}} |$). Adding springs reduces the mean($\Sigma | \Delta \text {PE}_{\text {T}} |$) by 98% for orientations $70^{\circ }<\psi <105^{\circ }$, from 128.7 to 2.65 N-m (Figure S8(C)). Figure 4B shows the plot of the potential energy contributions for $\psi = 90$. With the structure optimized for continuous equilibrium, the external mass is now counterbalanced both during reconfiguration of the knee joint and as the structure reorients about the ankle joint.

The three-dimensional origami arch is made from a variation of the Miura-ori unit cell, which is the base for many origami structures^52^. The arch is a single DOF mechanism consisting of sixty-four origami panels that fold from a flat state, with kinematics defined by the fold angle $\phi$ (Fig. 4C)^53^. We model the structure with a uniform mass distribution of 1 kg/m$^2$ and panel areas of approximately 0.1 m$^2$ (Figure S9). To keep the design simple, we limit possible spring connection points to locations within each unit cell. The optimization suggests using three internal torsional springs at the fold lines of the pattern ($\theta _A$, $\theta _B$, and $\theta _C$) and two internal extensional springs on each cell. The $\Sigma | \Delta \text {PE}_{\text {T}} |$ is reduced by 96.1%, from 43.0 to 1.69 N-m as it deploys from $\phi = 175^{\circ }$ to $100^{\circ }$. This example demonstrates that the principles from our work can be readily extended to an arbitrary three-dimensional system. While we limit the design to springs internal to each unit cell, the arch structure could be optimized using springs that interconnect any of the sixty-four panels. All of the possible spring connection points could be explored using a method similar to the ground structure approach used in topology optimization^54^.

**Figure 5:**
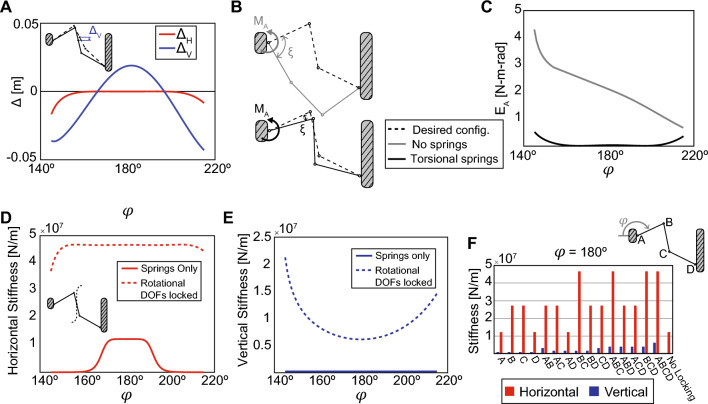
Considerations for the practical implementation of continuous equilibrium systems. (**A**) Residual displacements may occur, even with springs optimized for continuous equilibrium. For an idealized and frictionless Watt’s linkage, horizontal residual displacements are nearly zero for most of the kinematic path, and vertical residual displacements are small, but may be positive or negative from a desired configuration. (**B**) An external actuator applies a moment at location A on the Watt’s linkage. The angle $\xi$ describes the difference between the desired configuration (dashed lines) and the equilibrium position (due to gravity (gray solid lines) or gravity + springs (black solid lines)). (**C**) The energy in the actuator is nearly zero for the case with optimized torsional springs. (**D**) The Watt’s linkage with springs has stiffness in the horizontal direction near the center of the kinematic path, where the midpoint of the floating bar traces a straight line. Towards the ends of the kinematic path, the stiffness nears zero. When all of the rotational DOFs are locked, the Watt’s linkage has increased stiffness in the horizontal direction throughout the entire kinematic path. (**E**) The stiffness in the vertical direction increases by several orders of magnitude when all rotational DOFs are locked. (**F**) Locking the rotational DOFs at location combinations BC, ABC, or BCD all achieve the same horizontal stiffness as locking at all locations (ABCD). In the vertical direction, locking more rotational DOFs increases the stiffness.

### Considerations for practical implementation

We envision that the optimization method proposed in this paper can be used to design large-scale deployable and reconfigurable structures with reduced energy needed for actuation. However, aspects beyond the potential energy curve need to be considered to inform the practical implementation of these systems. In this section, we explore the Watt’s linkage to study residual displacements in the optimized systems, the reduction in actuation energy, and the influence of locking once the system reaches a desired state. A variety of methods can be used for the structural analysis of linkages^55^ and here we take a traditional structural engineering approach^56^.

#### Residual displacements

Even when springs with optimized parameters are placed on a system, the combined effects of gravity and spring forces may be imbalanced, leading to residual displacements (Fig. 5A). To calculate these potential residual displacements, we use a traditional structural engineering approach, constructing a stiffness matrix $[{\textbf{K}}]$ that represents the structure and solving for displacements {$\delta$} = $[{\textbf{K}}]^{-1}${F}, where {F} is the vector of applied loads, consisting of member self-weight and torsional spring moments (see Supporting Information for details). Displacements are measured at the midpoint of the floating link and remain less than 10% of member length for any point along the kinematic path. For this idealized system, the only component that resists the residual displacements is the stiffness of the springs. In practical scenarios, these residual displacements would be much lower due to friction in the system; for example, the physical prototype of the Watt’s linkage at $\psi = 0^{\circ }$ had minimal residual displacements when reconfigured along the kinematic path (Movie S2). In the next two subsections, we show that despite the internal force imbalance, there is still a major reduction in the energy required for actuation, and that locking serves as a practical approach to adding stiffness and preventing these residual displacements.

#### Reduced energy for actuation

We use the stiffness matrix formulation to explore the Watt’s linkage when a torsional actuator placed at location A is used to move the structure through its kinematic path. The actuator applies a moment $M_A$ to the structure in order to rotate it by an angle $\xi$, which is the angle between the equilibrium configuration (due to gravity and spring forces) and the desired configuration (defined by $\phi$) (Fig. 5B). We compare the energy in the actuator for the case with no springs and the case with four internal torsional springs (with properties found using optimization). The energy in the actuator $E_A$ is equal to $M_A$ multiplied by $\xi$. For the case of the Watt’s linkage with four internal torsional springs, $E_A$ is nearly zero for the entire kinematic path (Fig. 5C). Larger actuation moments are required at the ends of the kinematic path where the linkage no longer traces a vertical line, a similar trend to that shown in experimental results shown in Fig. 2G. This result considers that the actuator must overcome both the residual displacements and the stiffness of the internal springs when reconfiguring the structure. Similar trends are seen for the Watt’s linkage at $\psi = 45^{\circ }$ (Figure S12).

#### Locking

In reality, the optimized continuous equilibrium structures will remain flexible (similar to a mechanism) and locking of the system would be necessary to provide stiffness for functional load-bearing applications. The structural stiffness of the Watt’s linkage with internal torsional springs is computed using the stiffness matrix formulation where a unit force is applied in the middle of the linkage. Without locking, the linkage only has high stiffness in the horizontal direction at the center of the kinematic path, where the midpoint of the floating bar traces a vertical path (Fig. 5D, Figure S13(A)). In the vertical direction, the linkage is overly flexible and locking the four rotational joints (DOFs) increases the stiffness by several orders of magnitude (Fig. 5E, Figure S13(B)). The large increase in stiffness from joint locking occurs because the individual members can now carry loads in bending (sensitivity of stiffness to member moment of inertia in Figure S13(C-D)). The number and combination of locked nodes will also affect the structural stiffness. In the horizontal direction, locking locations BC, ABC, and BCD all result in the same stiffness as locking at all locations (Fig. 5F). In the vertical direction, locking more nodes always leads to a higher stiffness, although the structure remains more flexible than in the horizontal direction. While the stiffness of locking combinations change slightly for different configurations, the general trends stay the same (Figure S14). Similar trends are seen for the Watt’s linkage at $\psi = 45^{\circ }$ and $\psi = 90^{\circ }$ (Figure S15, Figure S16).

## Methods

More details on the computational methods and physical experiments are included in the Supplementary Materials.

### Computational methods

The optimization problems were solved using the function **fmincon** in MATLAB. The results presented in the ‘Considerations for Practical Implementation’ section are based on a formulation of a structural stiffness matrix where torsional springs are added at one end of classical beam elements.

### Experiments

Physical prototypes of the Watt’s linkage and Scissor Mechanism were fabricated as proof-of-concept continuous equilibrium systems. Linkage members were cut from acrylic sheets and have dimensions of 0.3048 m (12″) by 0.0381 m (1.5″) with a thickness of 8.4 mm (0.329″) for the Watt’s linkage and 16.2 mm (0.638″) for the Scissor Mechanism). A low-friction frame was used to support the Scissor Mechanism. Torsional springs were purchased from McMaster-Carr and attached using acrylic pieces. The reconfiguration force was measured using a load cell.

## Conclusions

In this paper, we introduce a comprehensive method for designing reconfigurable structures that maintain continuous equilibrium. Our method involves optimizing the properties of internal, external, torsional, and extensional springs that counteract gravity to minimize the fluctuation of the potential energy curve throughout the kinematic path. The optimization framework is extended to optimize structures for a range of orientations, leading to one design that has continuous equilibrium properties even as the orientation of the structure changes. Combinations of springs with asymmetric kinematics tend to result in better performance, and external springs are the most effective when considering a structure at multiple orientations. We demonstrated how our design framework can be applied to real-world systems including a linkage with an external mass carried along a linear path, a linkage with a mass carried along a radial path, and a three-dimensional deployable origami arch. Using computational simulations and physical proof-of-concept prototypes, we show that the proposed continuous equilibrium structures enable more efficient actuation. Using optimization to design for continuous equilibrium results in reconfigurable structures that are more stable, efficient, and versatile for any application scenario. The framework presented in this work will expand the ability of designers and engineers to create multi-functional systems to be used in robotics, infrastructure, consumer products, architecture, and more.

## Supplementary Information

Supplementary Movie 1.

Supplementary Movie 2.

Supplementary Movie 3.

Supplementary Movie 4.

Supplementary Movie 5.

Supplementary Movie 6.

Supplementary Movie 7.

Supplementary Movie 8.

Supplementary Information.

## Data Availability

All data generated or analyzed during this study are included in this published article, its supplementary information files, and through the online repository available at https://github.com/mariared-DRSL/Continuous-equilibrium-examples.
