# Supplementary material for: Designing continuous equilibrium structures that counteract gravity in any orientation
Source: Sci Rep. 2023 May 17;13:8007. doi: 10.1038/s41598-023-34760-1 (PMC10192317; doi:10.1038/s41598-023-34760-1)
Supplement: Supplementary file 9 — Supplementary Information. [file 41598_2023_34760_MOESM9_ESM.pdf]

Supplementary Information for: Designing continuous equilibrium structures that counteract gravity in any orientation

Maria Redoutey and Evgueni Filipov*

Optimized Spring Parameters

This section provides the spring parameters (stiffness and rest angle or rest length) found using optimization that lead to a continuous equilibrium system under gravity. All values were computed assuming the following linkage properties: member length = 0.3 m, uniform mass distribution = 1 kg/m, and cross-sectional area = 0.0254 m^2 unless otherwise noted.

Watt's Linkage at $\psi = 0°$

Internal torsional springs were placed at up to four locations, labelled A, B, C, and D in Figure 1. Table S1 contains the optimized rest angles α_j, stiffnesses k_j, and $\Sigma|\Delta \mathrm{PE_T}|$ values for all internal torsional spring location combinations for the Watt's Linkage at $\psi = 0°$. For the combinations of BCD and ABCD, the results are nearly equivalent. The variation between the two cases is likely due to the arbitrary initial optimization point, convergence threshold, and the bounds placed on the design parameters. With small changes to the optimization parameters, it could be shown that these two combinations lead to equivalent results. Changes to the optimization parameters would slightly influence the final variables and $\Sigma|\Delta \mathrm{PE_T}|$, but the overall trends and conclusions presented in the results would be preserved.

Watt's Linkage at orientations $\psi = 0°$ to $90°$

When considering orientations $\psi = 0°$ to $90°$, we explored three cases: four internal torsional springs, one external torsional spring, and both four internal and one external torsional spring. Spring parameters were found by minimizing the mean($\Sigma|\Delta \mathrm{PE_T}|$). For the case with no springs, the mean($\Sigma|\Delta \mathrm{PE_G}|$) = 1.382 N-m. Table S2 contains the spring properties and mean($\Sigma|\Delta \mathrm{PE_T}|$) values for all three cases.

*filipov@umich.edu

Table S1: Spring properties and $\Sigma|\Delta \mathrm{PE_T}|$ values for all possible location combinations of internal torsional springs on the Watt's linkage, optimized for $\psi = 0°$.

	Rest Angle				Stiffness [N-m/rad]				
	α_A	α_B	α_C	α_D	k_A	k_B	k_C	k_D	$\Sigma\lvert\Delta \mathrm{PE_T}\rvert$ [N-m]
No Springs									2.07
A	145.2°				1.96				1.03
B		76.9°				1.02			1.58
C			159.2°				1.40		0.75
D				137.5°				1.93	0.73
AB	146.1°	115.8°			2.01	0.071			1.06
AC	145.2°		159.2°		1.32		0.87		0.27
AD	168.5°			138.3°	0.072			1.93	0.77
BC		88.6°	158.7°			1.17	1.44		0.21
BD		130.3°		137.5°		0.031		1.95	0.74
CD			158.8°	137.8°			0.73	1.33	0.32
ABC	159.8°	133.3°	156.7°		2.18	1.40	1.75		0.19
ABD	168.4°	130.2°		137.5°	0.014	0.030		1.94	0.74
ACD	145.2°		159.2°	153.4°	1.31		0.866	0.0084	0.27
BCD		141.4°	157.8°	138.7°		1.01	1.01	2.00	0.064
ABCD	198.9°	142.2°	158.2°	138.5°	0.396	1.18	1.23	2.04	0.065

Table S2: Spring properties and mean($\Sigma|\Delta \mathrm{PE_T}|$) values for the Watt's linkage, optimized for $\psi = 0°$ to $90°$.

Springs		Rest Angle					Stiffness [N-m/rad]					
Internal Torsional	External Torsional	α_A	α_B	α_C	α_D	α_E	k_A	k_B	k_C	k_D	k_E	Mean($\Sigma\lvert\Delta \mathrm{PE_T}\rvert$)
✓		202.3°	139.3°	155.3°	147.7°		3.01	3.69	3.49	2.61		0.578 N-m
	✓					22.1°					0.569	0.445 N-m
✓	✓	203.5°	143.9°	159.2°	137.3°	306.8°	4.64	5.66	5.50	6.68	1.10	0.137 N-m

The results can be improved if we optimize for a smaller range of ψ. Table S3 gives the spring properties and mean($\Sigma|\Delta \mathrm{PE_T}|$) values for the Watt's linkage, considering ranges $0°$ to $30°, 60°$, and $90°$.

Scissor Mechanism at $\psi = 0°$

The first case we considered for the Scissor Mechanism at $\psi = 0°$ is with up to four internal torsional springs at locations A, B, C, and D (Figure 3(A)). Table S4 contains the optimized rest angles α_j, stiffnesses k_j, and $\Sigma|\Delta \mathrm{PE_T}|$ values for all internal torsional location combinations for the Scissor Mechanism at $\psi = 0°$. Figure S1 illustrates the potential energy contributions of components for each location combination.

In addition to four internal torsional springs, we considered adding two internal extensional springs and one external extensional spring to the Scissor Mechanism (Figure 3(B-C)). Table S5 contains the spring parameters and $\Sigma|\Delta \mathrm{PE_T}|$ values for each of the cases. The ex-

Table S3: Spring properties and mean($\Sigma|\Delta PE_T|$) values for the Watt's linkage, optimized for various ranges of ψ.

	Rest Angle					Stiffness [N-m/rad]					
Range	α_A	α_B	α_C	α_D	α_E	k_A	k_B	k_C	k_D	k_E	Mean($\Sigma\lvert\Delta PE_T\rvert$)
0° to 90°	203.5°	143.9°	159.2°	137.3°	306.8°	4.64	5.66	5.50	6.68	1.10	0.137 N-m
0° to 60°	209.9°	144.0°	159.2°	137.3°	333.0°	4.12	5.16	5.02	6.48	0.77	0.0905 N-m
0° to 30°	214.3°	144.0°	159.3°	137.3°	355.4°	2.86	3.80	3.72	5.12	0.42	0.0602 N-m

Table S4: Spring properties and $\Sigma|\Delta PE_T|$ values for all possible location combinations of internal torsional springs on the Scissor Mechanism, optimized for $\psi = 0°$.

	Rest Angle				Stiffness [N-m/rad				
	α_A	α_B	α_C	α_D	k_A [N-m/rad]	k_B [N-m/rad]	k_C [N-m/rad]	k_D [N-m/rad]	$\Sigma\lvert\Delta PE_T\rvert$ [N-m]
No Springs									1.77
A	179.5°				0.36				0.202
B		179.5°				0.36			0.202
C			0.49°				0.36		0.202
D				0.49°				0.36	0.202
AB	179.0°	179.0°			0.18	0.18			0.206
AC	179.0°		0.98°		0.18		0.18		0.206
AD	179.0°			0.98°	0.18			0.18	0.206
BC		179.0°	0.98°			0.18	0.18		0.206
BD		179.0°		0.98°		0.18		0.18	0.206
CD			0.98°	0.98°			0.18	0.18	0.206
ABC	173.2°	173.2°	6.76°		0.13	0.13	0.13		0.255
ABD	173.2°	173.2°		6.76°	0.13	0.13		0.13	0.255
ACD	173.2°		6.76°	6.76°	0.13		0.13	0.13	0.255
BCD		173.2°	6.76°	6.76°		0.13	0.13	0.13	0.255
ABCD	178.1°	178.1°	1.93°	1.93°	0.09	0.09	0.09	0.09	0.214

tensional springs are labelled as spring 1, which spans one unit of the Scissor Mechanism (stiffness k_1, rest length L_1), spring 2, which spans the entire structure (stiffness k_2, rest length L_2), and external spring 3, which is connected to an external anchor point (stiffness k_3, rest length L_3).

Scissor Mechanism at orientations $\psi = 0°$ to $90°$

For the Scissor Mechanism at orientations $\psi = 0°$ to $90°$, we explored seven cases including internal, external, torsional, and extensional springs. Table S6 contains the spring properties and mean($\Sigma|\Delta PE_T|$) for all cases.

The shape of the gravity curve changes significantly as the Scissor Mechanism is reoriented (Figure S2). As a result, the internal springs are less effective than the external springs at minimizing the mean($\Sigma|\Delta PE_T|$), because their potential energy contributions do not change with ψ. Furthermore, as shown in Figure 3 and Table S2, the optimal result for the mean($\Sigma|\Delta PE_T|$) can be substantially improved if all springs are used together.

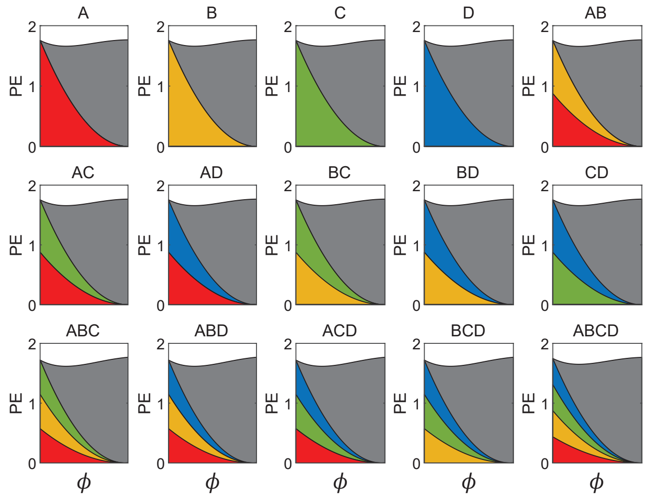

Figure S1: Potential energy breakdowns for all possible location combinations of internal torsional springs on the Scissor Mechanism, optimized for $\psi = 0°$. Due to symmetry in the system geometry, the total potential energy curve is nearly equivalent for all cases.

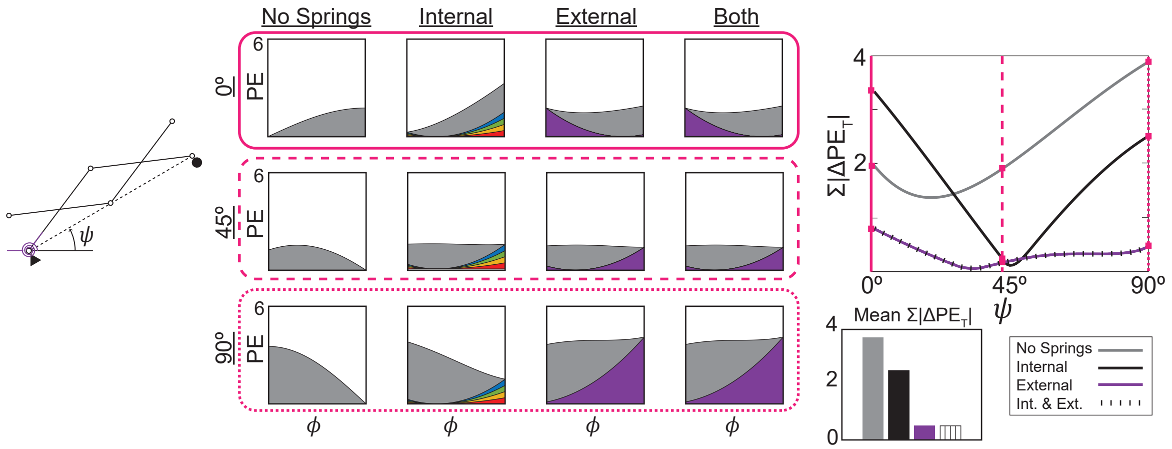

Figure S2: The potential energy due to gravity changes as the Scissor Mechanism is reoriented. As a result, the external torsional spring is more effective than the internal torsional springs at reducing the mean($\Sigma|\Delta PE_T|$). For the case with both internal and external torsional springs, the stiffness of each internal spring goes to zero and the system reverts to the case with one external torsional spring.

Table S5: Spring properties and $\Sigma|\Delta \mathrm{PE_T}|$ values for the Scissor Mechanism, optimized for $\psi = 0°$.

Springs			Rest Angle				Stiffness [N-m/rad]				Rest Length [m]			Stiffness [N/m]			
Int. Tor.	Int. Extens.	Ext. Extens.	α_A	α_B	α_C	α_D	k_A	k_B	k_C	k_D	L_1	L_2	L_3	k_1	k_2	k_3	$\Sigma\|\Delta \mathrm{PE_T}\|$
✓			178.1°	178.1°	1.93°	1.93°	0.09	0.09	0.09	0.09							0.214 N-m
	✓										0.503	0.024		11.66	4.125		0.004 N-m
		✓											1.26			10.6	0.0065 N-m

Table S6: Spring properties and mean($\Sigma|\Delta \mathrm{PE_T}|$) values for the Scissor Mechanism, optimized for $\psi = 0°$ to $90°$.

Springs				Rest Angle					Stiffness [N-m/rad]					Rest Length [m]			Stiffness [N/m]			
Int. Tor.	Ext. Tor.	Int. Extens.	Ext. Extens.	α_A	α_B	α_C	α_D	α_E	k_A	k_B	k_C	k_D	k_E	L_1	L_2	L_3	k_1	k_2	k_3	Mean($\Sigma\|\Delta \mathrm{PE_T}\|$)
✓				54.0°	54.0°	126.0°	126.0°		0.157	0.157	0.157	0.157								1.41 N-m
	✓							71.3°								2.28				0.287 N-m
✓	✓			89.7°	89.7°	90.3°	90.3°	71.3°	0	0	0	0	2.28							0.287 N-m
		✓												1.12	0.647		2.96	31.5		1.5378 N-m
			✓													1.46			15.5	0.794 N-m
✓		✓		45.3°	45.3°	134.7°	134.7°		0.133	0.133	0.133	0.133		0.125	0.326		11.5	3.88		1.41 N-m
	✓		✓					67.1°					2.21			0.754			2.76	0.232 N-m
✓	✓	✓	✓	82.7°	82.7°	97.3°	97.3°	31.9°	0	0	0	0	0.0063	0	3.44	0	26.7	0.0031	8.92	0.0013 N-m

Table S7: Spring properties and $\Sigma|\Delta PE_T|$ values for all possible location combinations of internal torsional springs on the Double Rocker linkage, optimized for $\psi = 0°$.

	Stiffness [N-m/rad]				Rest Angle				
	α_A	α_B	α_C	α_D	k_A [N-m/rad]	k_B [N-m/rad]	k_C [N-m/rad]	k_D [N-m/rad]	$\Sigma\|\Delta PE_T\|$ [N-m]
No Springs									0.372
A	101.6°				0.91				0.0598
B		136.8°				0.19			0.073
C			69.6°				0.20		0.0998
D				100.1°				0.98	0.073
AB	92.9°	172.3°			0.66	0.069			0.018
AC	97.8°		45.9°		0.702		0.058		0.014
AD	97.4°			95.8°	0.53			0.46	0.004
BC		140.6°	124.7°			0.18	0.012		0.073
BD		91.2°		94.2°		0.049		0.75	0.045
CD			162.6°	94.0°			0.033	0.88	0.054
ABC	98.1°	107.9°	45.8°		0.70	0.0033	0.056		0.015
ABD	108.7°	132.3°		116.5°	0.50	0.042		0.41	0.003
ACD	109.4°		83.9°	120.2°	0.57		0.048	0.31	0.002
BCD		95.6°	118.0°	94.2°		0.048	0.0039	0.74	0.046
ABCD	111.4°	129.8°	86.9°	122.4°	0.53	0.023	0.028	0.35	0.003

Double Rocker linkage

The Double Rocker linkage is a four-bar linkage with unequal bar lengths. The lengths of the input link, floating link, output link, and ground link are 0.25 m, 0.1 m, 0.3, and 0.2 m, respectively.

The Double Rocker linkage has four angles with kinematic paths that are not symmetric nor linearly related (Figure S4). When fitted, the orders of the potential energy contributions are 2nd, 3rd, 4th, and 3rd order for springs A, B, C, and D, respectively. This variety of higher order terms in PE_T gives the system more freedom to offset PE_G and leads to a more effective minimization of $\Sigma|\Delta PE_T|$.

Adding four internal torsional springs to the Double Rocker linkage reduces the $\Sigma|\Delta PE_T|$ by over 99%, from 0.372 N-m to 0.003 N-m. Table S7 contains the optimized rest angles α_j, stiffnesses k_j, and $\Sigma|\Delta PE_T|$ values for all location combinations for the Double Rocker linkage. Figure S3 illustrates the potential energy contributions of components for each location combination.

Similarly to the Scissor Mechanism, we consider adding extensional springs to the Double Rocker linkage (Figure S4(C-D)). For $\psi = 0°$, internal or external extensional springs reduce the mean($\Sigma|\Delta PE_T|$) by 99.5% and 92%, respectively.

We can also explore which type of springs are most effective when the Double Rocker linkage is reoriented between $\psi = 0°$ to 90°. For the system with only torsional springs, the combination of internal and external torsional springs reduces the fluctuation in potential energy the most (Figure S4(E)). Due to the lack of symmetry in the kinematics, the internal torsional springs have an effect, unlike the Scissor Mechanism. The case with external torsional and external extensional springs reduces the mean($\Sigma|\Delta PE_T|$) nearly as much as the case with all springs (Figure S4(E-F)); note the log scale and bar graph in Figure S4(F)). Table S9 contains the spring parameters for all spring cases of the Double Rocker linkage for orientations $\psi = 0°$ to 90°.

Table S8: Spring properties and $\Sigma|\Delta \mathrm{PE_T}|$ values for the Double Rocker linkage, optimized for $\psi = 0°$.

Springs			Rest Angle				Stiffness [N-m/rad]				Rest Length [m]			Stiffness [N/m]			
Int. Tor.	Int. Extens.	Ext. Extens.	α_A	α_B	α_C	α_D	k_A	k_B	k_C	k_D	L_1	L_2	L_3	k_1	k_2	k_3	$\Sigma\|\Delta \mathrm{PE_T}\|$
✓			111.4°	129.8°	86.9°	122.4°	0.53	0.02	0.03	0.35							0.003 N-m
	✓										0.30	0.27		19.8	21.1		0.0018 N-m
		✓											1.20			38.6	0.030 N-m

Table S9: Spring properties and mean($\Sigma|\Delta \mathrm{PE_T}|$) values for the Double Rocker linkage, optimized for $\psi = 0°$ to $90°$.

Springs				Rest Angle					Stiffness [N-m/rad]					Rest Length [m]			Stiffness [N/m]			
Int. Tor.	Ext. Tor.	Int. Extens.	Ext. Extens.	α_A	α_B	α_C	α_D	α_E	k_A	k_B	k_C	k_D	k_E	L_1	L_2	L_3	k_1	k_2	k_3	Mean($\Sigma\|\Delta \mathrm{PE_T}\|$)
✓				199.9°	175.4°	76.5°	161.0°		0.90	0.04	0.001	0.003								0.219 N-m
	✓							67.9°					0.55							0.150 N-m
✓	✓			90.1°	93.2°	81.5°	152.2°	46.5°	0.012	0.043	0.044	0.014	0.434							0.088 N-m
		✓												1.86	0.20		1.66	18.3		0.185 N-m
			✓													1.24			10.1	0.199 N-m
✓		✓		84.1	93.4	105.0	145.5		0.047	0.024	0.077	0.075		0.19	0.13		2.54	28.1		0.185 N-m
	✓		✓					40.6					0.44			1.01			91.3	0.0941 N-m
✓	✓	✓	✓	90.5	53.3	135.8	136.9	125.1	6.91	0.27	0.18	1.11	0.43	0.06	0.35	0.27	48.3	33.0	45.6	0.089 N-m

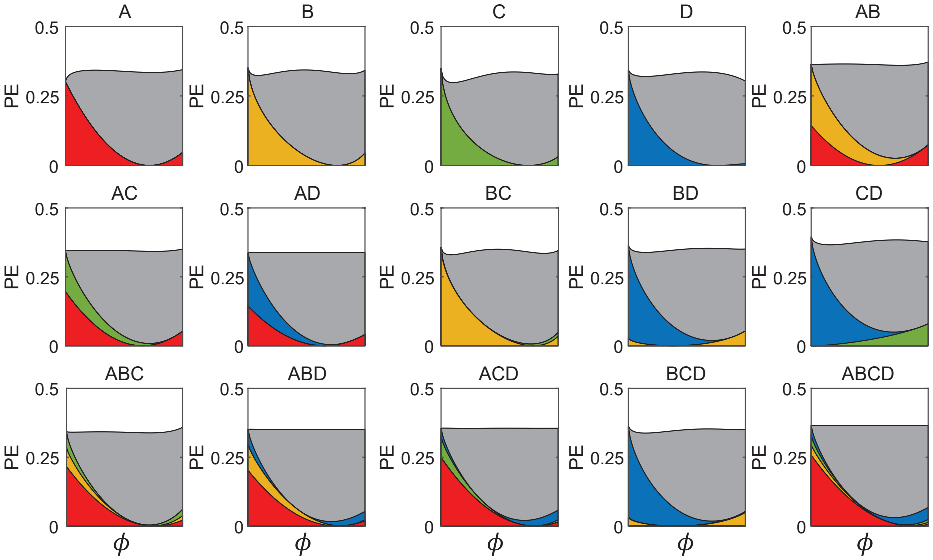

Figure S3: Potential energy breakdowns for all possible location combinations of four internal torsional springs on the Double Rocker linkage, optimized for $\psi = 0°$.

Curve Fitting of Spring Kinematic Relationships

We use the MATLAB function **fit** to determine the order of the spring angle curves $\theta_A, \theta_B, \theta_C, \theta_D$ when they are plotted against the kinematic angle ϕ. We consider polynomial fits with orders 1 (linear), 2 (quadratic), 3 (cubic), and 4 (quartic). We use the coefficient of determination (R^2 value) to determine the polynomial that best fits the kinematic curve. The maximum possible R^2 value is 1.

The angle kinematics for the Watt's linkage are plotted in Figure 1. We define θ_A as equal to ϕ, so a linear fit provides an R^2 value of 1. The angles θ_B, θ_C, and θ_D require fourth-order fits to reach an R^2 value of 1.

Table S10: R^2 values for polynomial Watt's Linkage angle kinematics.

Fit Type	θ_A	θ_B	θ_C	θ_D
Linear	1	0.8935	0.8738	0.9946
Quadratic		0.9989	0.9970	0.9990
Cubic		0.9998	0.9987	0.9993
Quartic		1	1	1

The kinematics of the Scissor Mechanism are plotted in Figure 3. Due to symmetry in the system geometry, there are two sets of symmetric angles which are linearly related to ϕ and to each other. As a result, all of the angle curves have an R^2 value of 1 for a linear

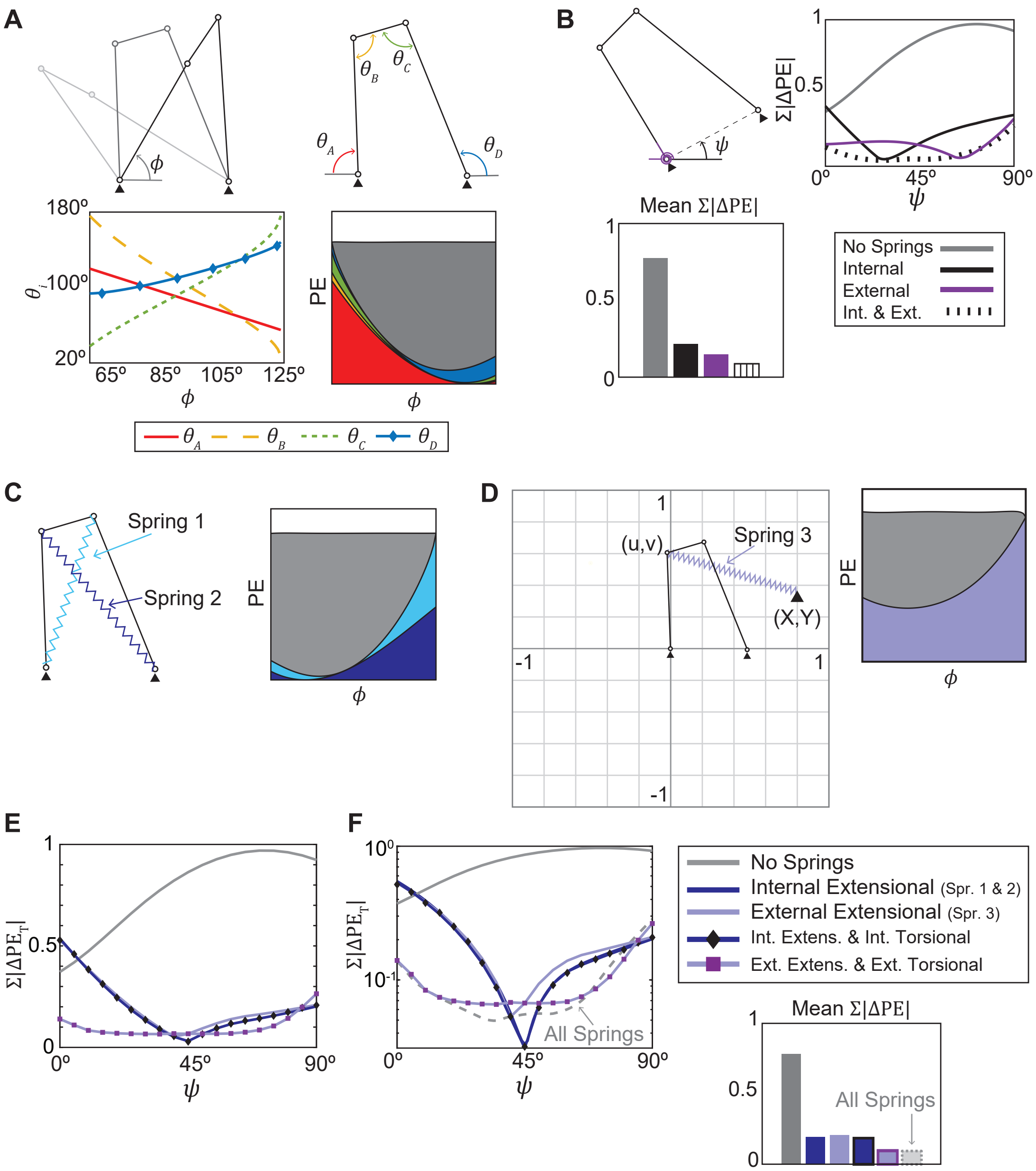

Figure S4: (A) The Double Rocker linkage is a four-bar linkage with three links of unequal lengths. Four locations for internal torsional springs are defined with angles θ_A, θ_B, θ_C, and θ_D. There is no symmetry in the spring angle kinematics. Adding four internal torsional springs reduces the $\Sigma|\Delta PE_T|$ by over 99%. (B) When considering only torsional springs, adding both internal and external torsional springs is the most effective for reducing the mean($\Sigma|\Delta PE_T|$). (C) Adding two internal extensional springs to the Double Rocker linkage reduces the $\Sigma|\Delta PE_T|$ by 99.5%. (D) Using an external extensional spring reduces the $\Sigma|\Delta PE_T|$ by 92%. (F) Using a combination of all springs marginally reduces the mean($\Sigma|\Delta PE_T|$) from the case with external torsional and extensional springs.

fit. As discussed in the main text, because the kinematics of all springs are linearly related, the number and placement of the springs does not have a significant effect on the optimized potential energy behavior.

Table S11: R^2 values for polynomial fits of Scissor Mechanism angle kinematics.

Fit Type	θ_A	θ_B	θ_C	θ_D
Linear	1	1	1	1
Quadratic				
Cubic				
Quartic				

The lengths of the internal extensional springs (l_1 and l_2) are not linearly related to ϕ or to each other. They can be fitted using polynomial and sinusoidal functions. The length of extensional spring 1 is directly related to ϕ: $l_1 = L \sin\phi$, where L is the member length, so a one-term sinusoidal fit results in an R^2 value of 1. The length of extensional spring 2 requires a three-term sinusoidal fit for an R^2 value of 1.

Table S12: R^2 values for various fits of Scissor Mechanism internal extensional spring lengths.

Fit Type	l_1	l_2
Linear	0.9575	0.9811
Quadratic	0.9992	0.9874
Cubic	1.0000	0.9996
Quartic		1.0000
Sinusoidal (1-term)	1.0000	0.9886
Sinusoidal (2-term)		0.9992
Sinusoidal (3-term)		1.0000

Table S13: R^2 values for sinusoidal fits of Scissor Mechanism internal extensional spring lengths.

Fit Type	l_1	l_2
Sinusoidal (1-term)	1.0000	0.9886
Sinusoidal (2-term)		0.9992
Sinusoidal (3-term)		1.0000

The kinematics of the Double Rocker linkage are plotted in Figure S4. The angle θ_A is linearly related to ϕ: $\theta_A = \pi - \phi$, so the R^2 value for the linear fit is equal to 1. The angles θ_B and θ_C have third-order (cubic) fits with respect to ϕ, and θ_D has a fourth-order (quartic) fit.

Table S14: R^2 values for polynomial fits of Double Rocker angle kinematics.

Fit Type	θ_A	θ_B	θ_C	θ_D
Linear	1	0.9965	0.9916	0.9792
Quadratic		0.9965	0.9949	0.9992
Cubic		0.9995	0.9987	0.9993
Quartic		1.0000	1.0000	1.0000

Table S15: R^2 values for sinusoidal fits of Double Rocker internal extensional spring lengths.

Fit Type	l_1	l_2
Sinusoidal (1-term)	0.9992	1.0000
Sinusoidal (2-term)	0.9998	
Sinusoidal (3-term)	1.0000	

Physical Prototypes and Testing of the Watt's Linkage

We fabricated three versions of the Watt's linkage: one with no springs, one with four internal torsional springs, and one with four internal and one external torsional spring. The members of the linkages were fabricated using acrylic sheets with thickness = 2.7 mm (0.106"), length = 0.3048 m (12") and width = 0.0381 m (1.5"). The sheets were glued together to create members with a total thickness of 0.0081 m (0.329"). Additional acrylic pieces were used to attach the springs. Members were connected using bolts and nuts.

The spring parameters for the model with four internal torsional springs are presented in Table S16. The spring parameters for the model with four internal torsional springs and one external torsional spring are presented in Table S17. The tables provide both the calculated values (found using optimization) and the actual parameters of the springs used in fabrication. For the springs at locations B, C, and D, two springs of equal stiffness were used to create a composite spring with the total stiffness needed (Figure S5(B)). Despite using springs with some deviation from the calculated parameters, our results show that the system with springs exhibits continuous equilibrium properties, where the system remains stable in different configurations and in different orientations (Figure S5(D), Figure 1(D), Figure 2(F)).

We used a force gauge (load cell) to measure the reconfiguration force of the Watt's linkage along the kinematic path (Figure 2(G) in the main text). The load cell was attached to the Watt's linkage at location B and a vertical force (pulling or pushing) was applied. The force for the system with springs is nearly centered around 0 N, with higher forces developing at both ends of the kinematic path (positive force for pulling up, negative force for pushing down), where the linkage deviates from a straight vertical path. Overall, the forces for the system with springs are lower than the system without springs.

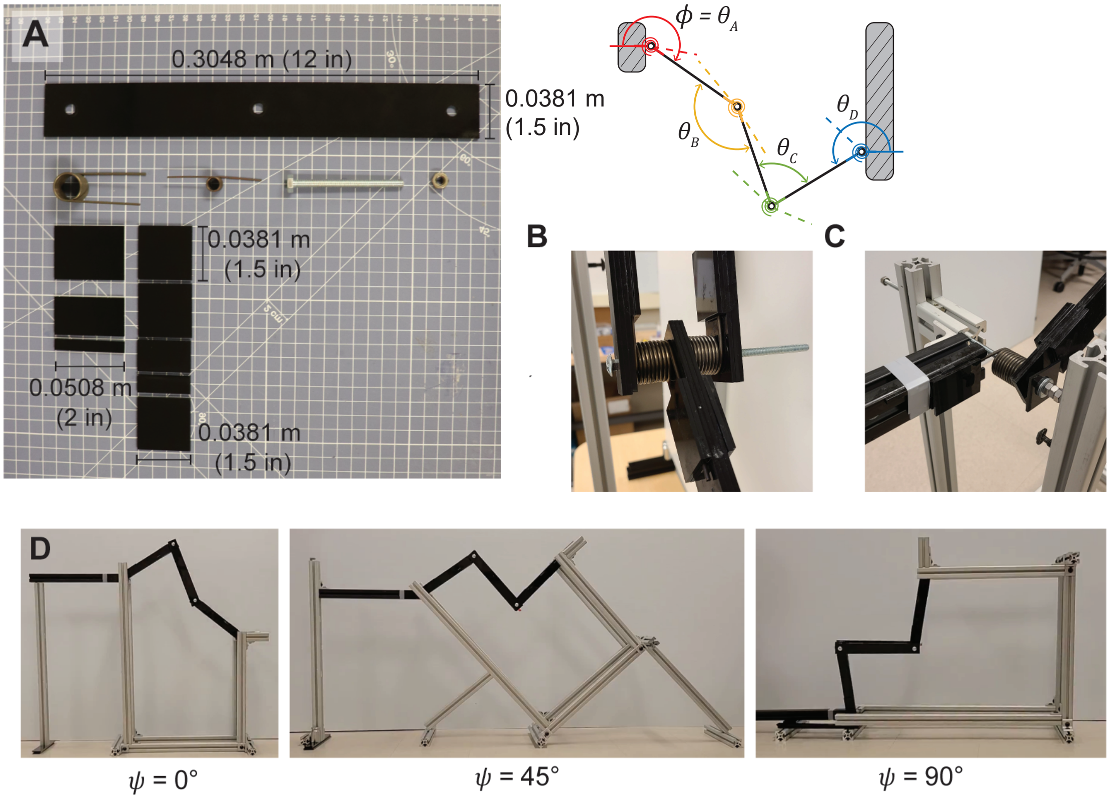

Figure S5: Physical prototype of the Watt's linkage. (A) Members of the Watt's linkage were cut from acrylic sheets. (B) Two springs were used at locations B, C, and D to achieve the required spring stiffness. (C) For the system that can be re-oriented, an external torsional spring was installed with one end connected to the Watt's linkage at location A and one end connected to a horizontal bar (shown in black). An internal spring was not used at location A, because it does not add a significant influence on the overall system behavior (see Figure 2(B)). (D) With internal and external torsional springs, the Watt's linkage can be reconfigured at $\psi = 0°$, $45°$, and $90°$ without collapsing.

Table S16: Spring properties (calculated and used values) for the physical model of the Watt's linkage with four internal torsional springs, optimized for $\psi = 0°$.

	Stiffness [N-m/rad]		Rest Angle	
Location	Calculated	Used	Calculated	Used
A	0.315	0.376	192°	200°
B	1.07	1.51	142°	135°
C	1.12	1.51	158°	135°
D	1.87	2.45	139°	135°

Table S17: Spring properties (calculated and used values) for the physical model of the Watt's linkage with four internal torsional springs and one external torsional spring, optimized for $0° \leq \psi \leq 90°$.

	Stiffness [N-m/rad]		Rest Angle	
Location	Calculated	Used	Calculated	Used
A	0.267	-	187°	-
B	1.87	1.51	144°	135°
C	1.64	1.51	159°	135°
D	2.48	2.45	137°	135°
External	1.04	1.22	191°	135°

Physical Prototypes and Testing of the Scissor Mechanism

We fabricated two models of the Scissor Mechanism: one without springs and one with four internal torsional springs. The members of the linkages were fabricated using acrylic sheets with thickness = 2.7 mm (0.106"), length = 0.3048 m (12") and width = 0.0381 m (1.5") (Figure S6(A)). The sheets were glued together to create members with a total thickness of 0.0162 m (0.638"). Additional acrylic pieces were used to attach the springs. Members were connected using bolts and nuts. A low-friction frame was built to support the linkages. The optimized stiffness of all of the springs is 0.09 N-m/rad, and we use springs with a stiffness of 0.1 N-m/rad in the physical model. The springs at locations A and C have a rest angle (optimized and used values) of 180° (Figure S6(B)) and two have a rest angle of 0° (locations B and D, Figure S6(C)).

The model without springs collapses due to gravity (Movie S7). The model with optimized springs can be reconfigured easily and is stable at any position along its kinematic path (Movie S8).

We measured the force needed to reconfigure the Scissor Mechanism using a load cell (Figure S6(D)). The forces required to reconfigure the model with springs are lower than those required to reconfigure the model without springs (Figure S6(E)). We can also calculate

the analytical solution for the horizontal force required to prevent the Scissor Mechanism from collapsing: $F_x = \frac{F_g}{2\tan\phi}$ (solid line in Figure S6(E)). The force gauge data matches this solution well for $45° \leq \phi \leq 90°$. For $\phi < 45°$, the force gauge method is not able to capture the increase in F_x, which tends towards infinity as $\phi \longrightarrow 0$. In the physical testing of the system with no springs, we have to reorient the load cell upward so that we can move the system, and this reorientation reduces our recorded force for $\phi < 45°$.

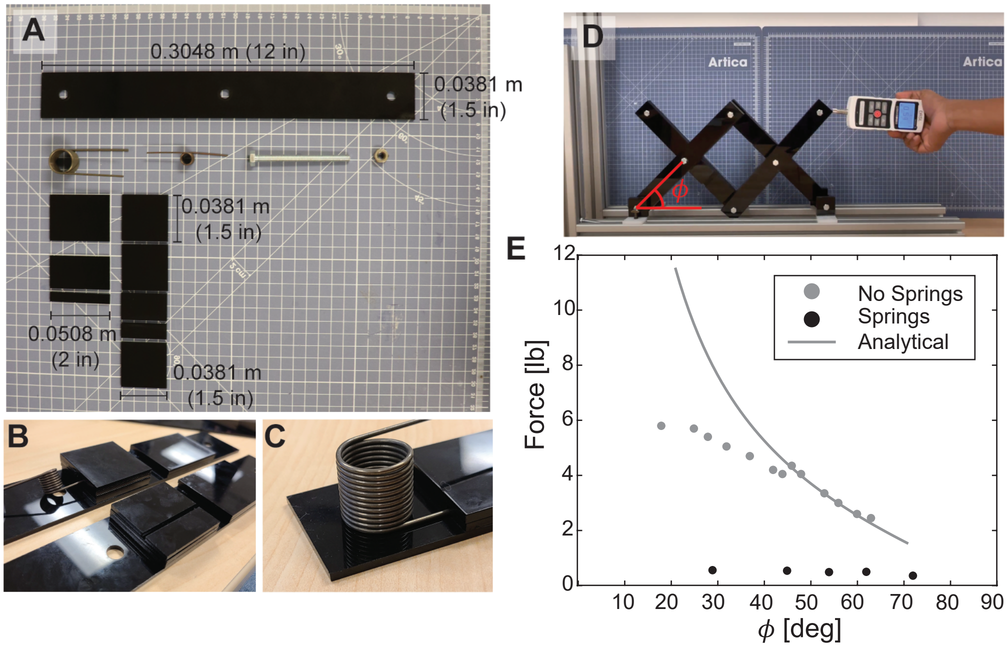

Figure S6: (A) Components of the Scissor Mechanism model. The members and attachment pieces are cut from acrylic sheets. The members are connected using bolts. (B) Spring attachment for locations A and C. (C) Spring attachment for locations B and D. (D) A load cell (force gauge) was used to measure forces required for reconfiguration.(E) Force measurements for the system with springs and without springs. The system with springs requires lower forces to reconfigure than the system with no springs. An analytical solution for the horizontal force required to maintain equilibrium matches the load cell data well for $\phi > 45°$ (solid line).

Additional Design Cases

Scissor Lift

The Scissor Lift is modeled using bar lengths of 1 m, uniform mass distribution of 10 kg/m, and an external load of 200 kg applied at the center of the fifth scissor unit (Figure S7). The optimized spring properties of the internal extensional springs placed at each unit of the structure (shown in light blue in Figure 4(A)) are k_1 = 4060.4 N/m and L_1 = 0.326 m. The properties of the internal extensional spring that spans the entire structure (shown in dark blue) are k_2 = 431.8 N/m and L_2 = 0.333 m. The parameters for the external

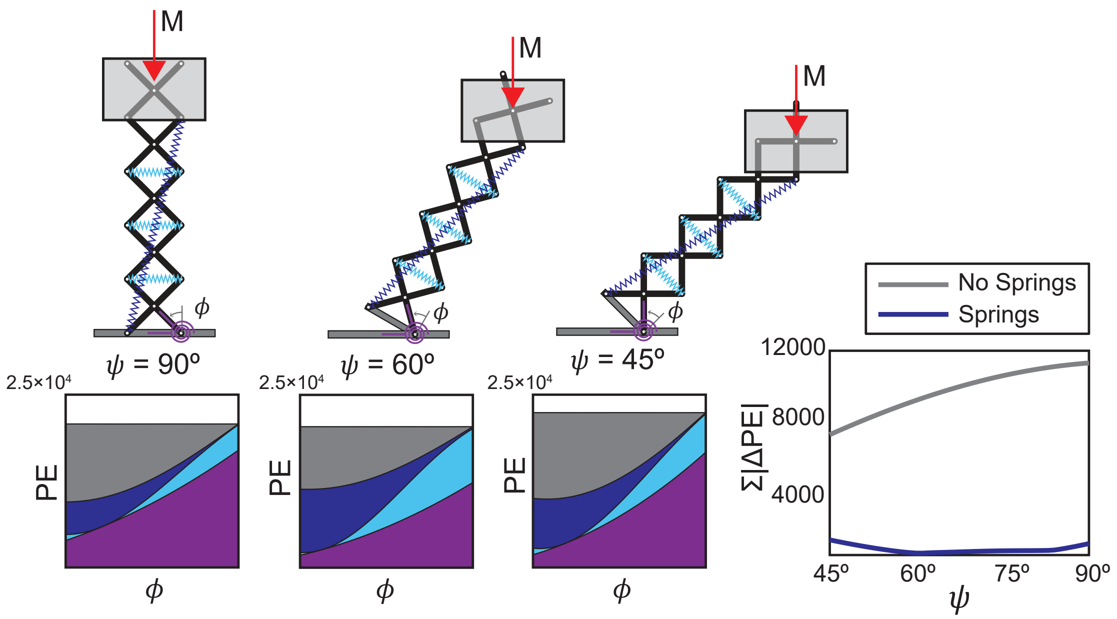

Figure S7: The Scissor Lift at multiple orientations: $\psi = 45°$, $\psi = 60°$, and $\psi = 90°$. The external torsional spring and internal extensional springs can be optimized to give the system continuous equilibrium for a range of orientations. For the range $\psi = 45°$ to $90°$, adding the internal extensional and external torsional springs greatly reduces the mean($\Sigma|\Delta \mathrm{PE_T}|$).

torsional spring are $k_\mathrm{E} = 3725.8$ N-m/rad and $\alpha_\mathrm{E} = 7.05°$. With the addition of springs, the fluctuation in the potential energy curve is reduced from 11282 N-m to 23.2 N-m.

Like the Scissor Mechanism, the scissor lift can be reoriented. For specific orientations, adding an external torsional spring and internal extensional springs successfully flatten the $\mathrm{PE_T}$ curve, as was shown for the case of $\psi = 90°$ in Figure 4(A). The curves for a system that is are optimized for $\psi = 45°$ to $\psi = 90°$ are shown in Figure S7. When considering a range of orientations $45° < \psi < 90°$, the $\Sigma|\Delta \mathrm{PE_T}|$ is significantly lower than for the case with no springs. The mean($\Sigma|\Delta \mathrm{PE_T}|$) for the case with no springs is 9640 N-m, and the mean($\Sigma|\Delta \mathrm{PE_T}|$) with springs is 336.2 N-m; a 96.5% reduction. The spring properties are as follows: $k_1 = 5043.5$ N/m, $L_1 = 0.235$ m, $k_2 = 639.8$ N/m, $L_2 = 0.284$ m, $k_E = 3665.0$ N-m/rad, $\alpha_E = 0°$.

Knee exoskeleton

We model a knee exoskeleton as two 45 cm members connected to a "foot" which is anchored to the ground (Figure S8(A)). A four-bar linkage (member length = 15 cm) with four internal torsional springs is placed at the knee joint. An internal extensional spring is connected to location B on the linkage and to the heel joint of the structure. The loads applied to the structure are the self weight of the members (2.6 kg per bar) and an external mass M = 30 kg. The internal torsional springs have kinematics that are symmetric and linearly related (Figure S8(B)) while the internal extensional spring has a sinusoidal relationship with ϕ.

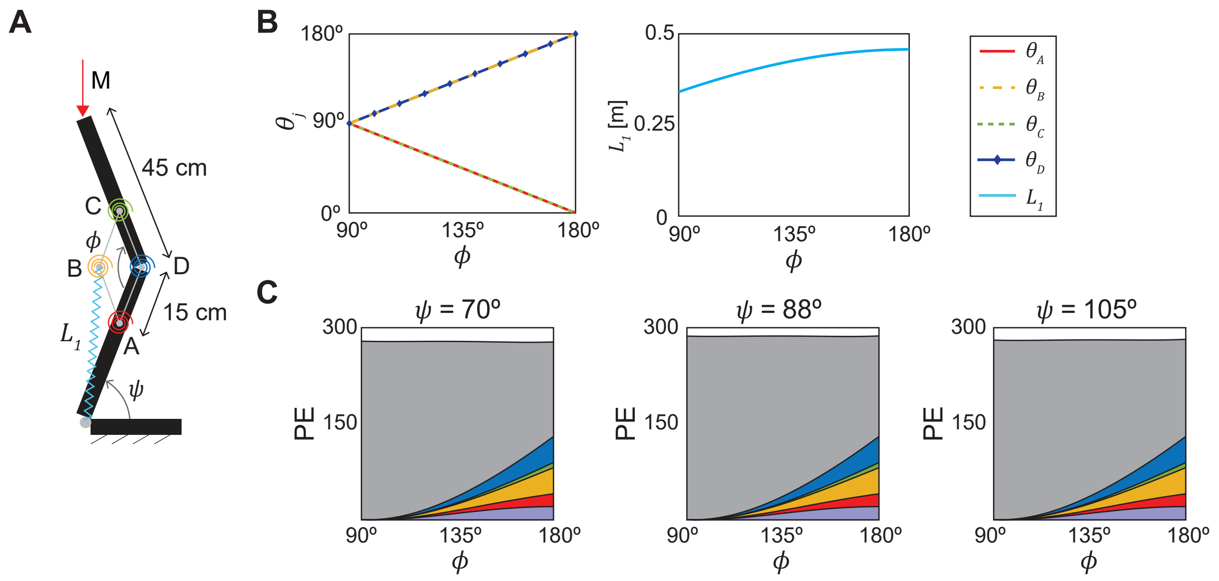

Figure S8: (A) The knee is modeled as two members connected to a "foot" which is anchored to the ground. A symmetric four-bar linkage is placed at the knee. (B) The internal torsional springs are linearly related to ϕ, while the internal extensional spring has a sinusoidal relationship with ϕ. (C) The magnitude of the potential energy due to gravity changes slightly for different orientations, but the overall shape of the curve remains constant. For orientations $70° \leq \psi \leq 105°$, internal springs flatten the potential energy curve.

The shape of the gravity curve stays constant as ψ changes, and its magnitude changes only slightly; therefore, internal springs are sufficient (Figure S8(C)). The optimized spring parameters for the knee model at $\psi = 70°$ to $105°$ are $\alpha_A = 88.3°$, $\alpha_B = 90.9°$, $\alpha_C = 86.1°$, $\alpha_D = 90.9°$, $k_A = 16.8$ N-m/rad, $k_B = 33.8$ N-m/rad, $k_C = 7.11$ N-m/rad, $k_D = 33.7$ N-m/rad, $L_1 = 0.354$ m, $k_1 = 3856.2$ N/m. Without springs, the mean($\Sigma|\Delta \mathrm{PE_G}|$) = 128.7 N-m. With springs, the mean($\Sigma|\Delta \mathrm{PE_T}|$) = 2.65 N-m.

Origami Arch

The origami arch is a three-dimensional structure based on a variation of the Miura-ori unit cell *(47)*. The arch deploys from a flat sheet. The deployment is defined by the kinematic angle ϕ; we consider a range of $100° < \phi < 175°$. The panel dimensions are shown in Figure S9(A). We optimized properties of three internal torsional springs and two internal extensional springs per unit cell. The optimized spring parameters are $\alpha_A = 140.1°$, $k_A = 0.3572$ N-m/rad, $\alpha_B = 123.9°$, $k_B = 0.6526$ N-m/rad, $\alpha_C = 81.0°$, $k_C = 1.443$ N-m/rad, $L_1 = 0.136$ m, $k_1 = 12.4$ N/m. The $\Sigma|\Delta \mathrm{PE_T}|$ is reduced from 43.0 N-m to 1.69 N-m. The kinematics of the fold angles are not symmetric or linearly related to each other, and the length of the extensional spring has a quadratic relationship with ϕ (Figure S9(B)). These factors result in the internal springs effectively minimizing the $\Sigma|\Delta \mathrm{PE_T}|$. While we have not optimized this system for reorientation, the origami arch could be rotated about a given axis and optimized for a range of orientations. When considering a range of orientations, we

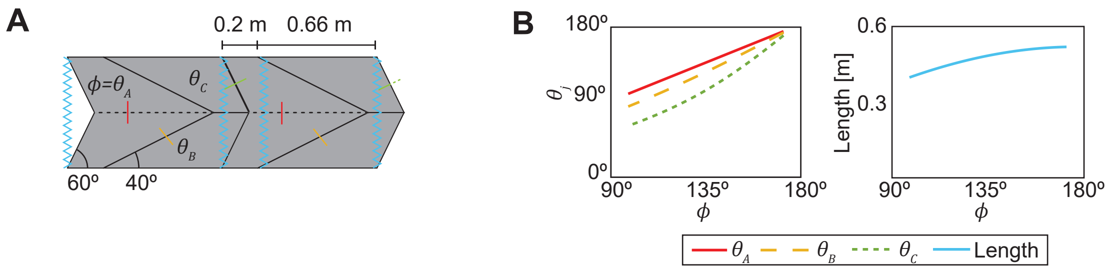

Figure S9: (A) The Miura-ori arch structure is made of an array of unit cells that are a variation of the Miura-ori pattern. Three internal torsional springs are added to the system at θ_A, θ_B, and θ_C, and two extensional springs are included in each cell. (B) The angles of the Miura-ori arch are not symmetric or linearly related. The length of the extensional spring is also not linearly related to the angles with respect to the kinematics defined by ϕ.

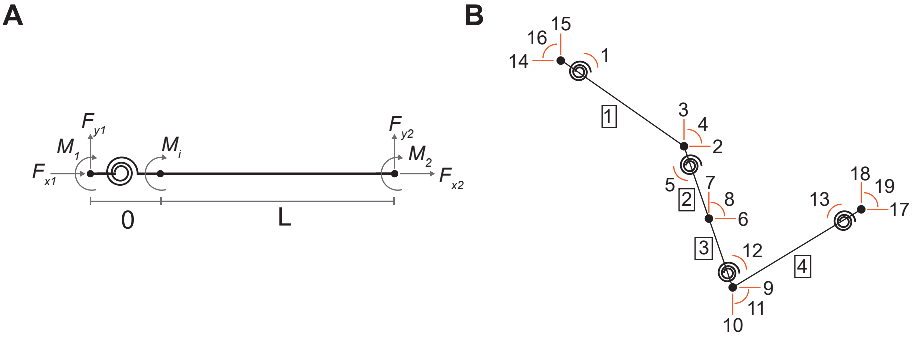

Figure S10: (A) To model the Watt's linkage with torsional springs, we use a frame member with an additional rotational degree of freedom (DOF) at one end. Each member has seven DOFs. (B) The DOFs for the Watt's linkage with four internal torsional springs. Members 1 and 2 have an additional rotational DOF at the i-node, and members 3 and 4 at the j-node.

expect that external springs would be needed.

Stiffness Matrix Formulation

To use the stiffness method to analyze the Watt's linkage with prestressed springs, we constructed a stiffness matrix $[\mathbf{K}]$ with additional rotational degrees of freedom (DOFs). The formulation of the stiffness matrix is an adaptation of the formulation for flexible connections presented in McGuire et al [56]. Figure S10(A) shows the DOFs for one member with a flexible connection at the i-node. For the Watt's linkage, we define four members, each modeled as a frame element with a spring at one end. The local stiffness matrix for each member is 7x7. We use a de-coupled approach so that moments can be applied at the added rotational degrees of freedom (DOFs 1, 5, 12, and 13 in Figure S10(B)), representing the pre-stress that is developed in the springs.

Member 1 in Figure S10(B) has a spring at its i-node with stiffness k_A. The local stiffness matrix for member 1 is then:

$$\begin{array}{cccccccc} \text{DOF}: & 14 & 15 & 16 & 1 & 2 & 3 & 4 \end{array}$$

$$[\hat{k_1}] = \begin{bmatrix} \frac{EA}{L} & 0 & 0 & 0 & -\frac{EA}{L} & 0 & 0 \\ 0 & \frac{12EI}{L^3} & 0 & \frac{6EI}{L^2} & 0 & -\frac{12EI}{L^3} & \frac{6EI}{L^2} \\ 0 & 0 & k_A & -k_A & 0 & 0 & 0 \\ 0 & \frac{6EI}{L^2} & -k_A & \frac{4EI}{L}+k_A & 0 & -\frac{6EI}{L^2} & \frac{2EI}{L} \\ -\frac{EA}{L} & 0 & 0 & 0 & \frac{EA}{L} & 0 & 0 \\ 0 & -\frac{12EI}{L^3} & 0 & -\frac{6EI}{L^2} & 0 & -\frac{12EI}{L^3} & -\frac{6EI}{L^2} \\ 0 & \frac{6EI}{L^2} & 0 & \frac{2EI}{L} & 0 & -\frac{6EI}{L^2} & \frac{4EI}{L} \end{bmatrix}$$

where E is the Young's Modulus, A is the member cross-sectional area, I is the moment of inertia, and L is the member length. For a member with a spring at its j-node, such as member 4, the additional DOF is included at that node. The local stiffness matrix for member 4 is then:

$$\begin{array}{cccccccc} \text{DOF}: & 9 & 10 & 11 & 17 & 18 & 19 & 13 \end{array}$$

$$[\hat{k_4}] = \begin{bmatrix} \frac{EA}{L} & 0 & 0 & -\frac{EA}{L} & 0 & 0 & 0 \\ 0 & \frac{12EI}{L^3} & \frac{6EI}{L^2} & 0 & -\frac{12EI}{L^3} & 0 & \frac{6EI}{L^2} \\ 0 & \frac{6EI}{L^2} & \frac{4EI}{L} & 0 & -\frac{6EI}{L^2} & 0 & \frac{2EI}{L} \\ -\frac{EA}{L} & 0 & 0 & \frac{EA}{L} & 0 & 0 & 0 \\ 0 & -\frac{12EI}{L^3} & -\frac{6EI}{L^2} & 0 & \frac{12EI}{L^3} & 0 & -\frac{6EI}{L^2} \\ 0 & 0 & 0 & 0 & 0 & k_D & -k_D \\ 0 & \frac{6EI}{L^2} & \frac{2EI}{L} & 0 & -\frac{6EI}{L^2} & -k_D & \frac{4EI}{L}+k_D \end{bmatrix}$$

These local stiffness matrices can then be assembled into the global stiffness matrix $[\mathbf{K}]$ and used to solve for the nodal displacements and rotations $\{\delta\} = [\mathbf{K}]^{-1}\{\mathrm{F}\}$, where $\{\mathrm{F}\}$ is a vector of applied loads. The external loads applied to the Watt's linkage are the gravity forces, which act downward at the center of mass of each of the bars, and the spring moments at each of the four spring locations. The gravity forces are equal to $-mg$, where m is the mass of a bar and $g = 9.81$ m/s^2 is the acceleration due to gravity. The moment at spring j is equal to $k_j(\theta_j - \alpha_j)$, where k_j and α_j are found using optimization. The applied moment changes as the linkage moves through its the kinematic path, reflecting the spring moving toward or away from its rest angle. The properties of the members are assumed to be $A = 0.0254$ m^2, $I = \frac{1}{12}(0.0254)(0.0254^3)$ m^4, and $E = 200$ GPa unless otherwise noted. The floating link is split into two members to give a DOF at the center of the linkage, so there are four members total. Members 1 and 4 have a length of 0.3 m, and members 2 and 3 have a length of 0.15 m.

Structural Stiffness of Watt's Linkage

To evaluate the stiffness of the Watt's linkage, we applied unit loads $P = 1.0$ to the centroid of the floating link, along with the gravity load and spring moments. Using the stiffness

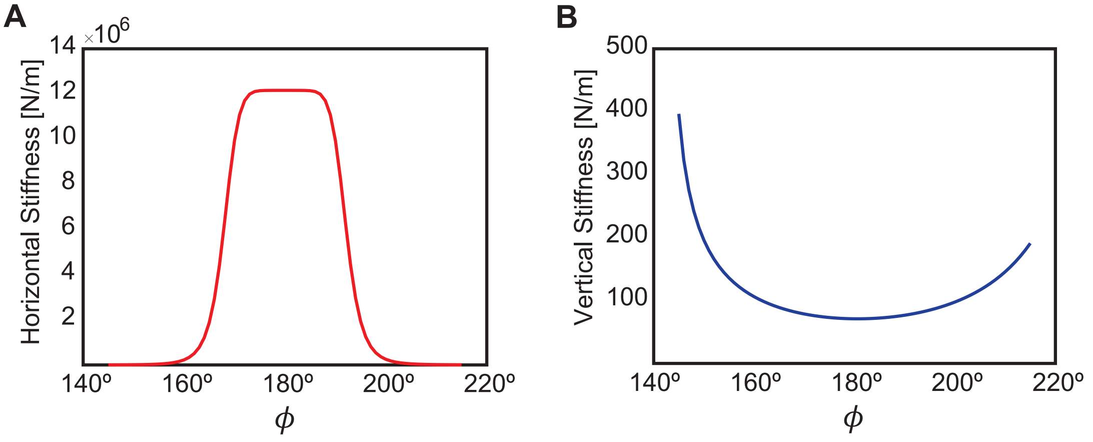

Figure S11: (A) Stiffness of the Watt's linkage in the horizontal direction (perpendicular to the kinematic path). The stiffness is largest at $\phi = 180°$. (B) Stiffness in the vertical direction (parallel to the kinematic path) is several orders of magnitude lower than in the horizontal direction.

method described, we then solved for the resulting displacements $\{\delta\}$ and calculated a structural stiffness equal to $\{\delta\}/\{F\}$.

Figures S11(A) and (B) show the structural stiffness of the Watt's linkage in the horizontal and vertical directions, respectively. With the addition of springs, the linkage has high stiffness for a load perpendicular to its kinematic path. For a load parallel to its kinematic path, the Watt's linkage has low stiffness, and thus is easily reconfigured. The Watt's linkage without torsional springs has no structural stiffness and collapses under gravity without locking. The springs are critical to the stiffness of the structure, more than the members of the linkage.

Residual Displacements and Actuation

Even with the addition of torsional springs with optimized parameters, the structure may experience some residual displacements in configurations where the gravity and spring forces do not directly offset. At $\psi = 0°$, the horizontal displacement is nearly zero throughout the entire kinematic path; the Watt's linkage is known to approximate vertical straight-line motion (Figure S12(A)). The vertical displacements are reflected in the rotation angle ξ (Figure S12(B)), which reflects the difference between the desired configuration angle ϕ and the equilibrium state of the structure due to the effects of gravity and springs. Adding springs reduces ξ significantly from the case with no springs (gray line in Figure S12(B)). The magnitude of the actuation moment M_A is comparable for the cases with and without springs (Figure S12(C)), but the actuation energy $E_A = M_A * \xi$ is nearly zero throughout the entire kinematic path when springs are added (Figure S12(D)).

We conduct the same study of the residual displacements, rotation angle, actuation mo-

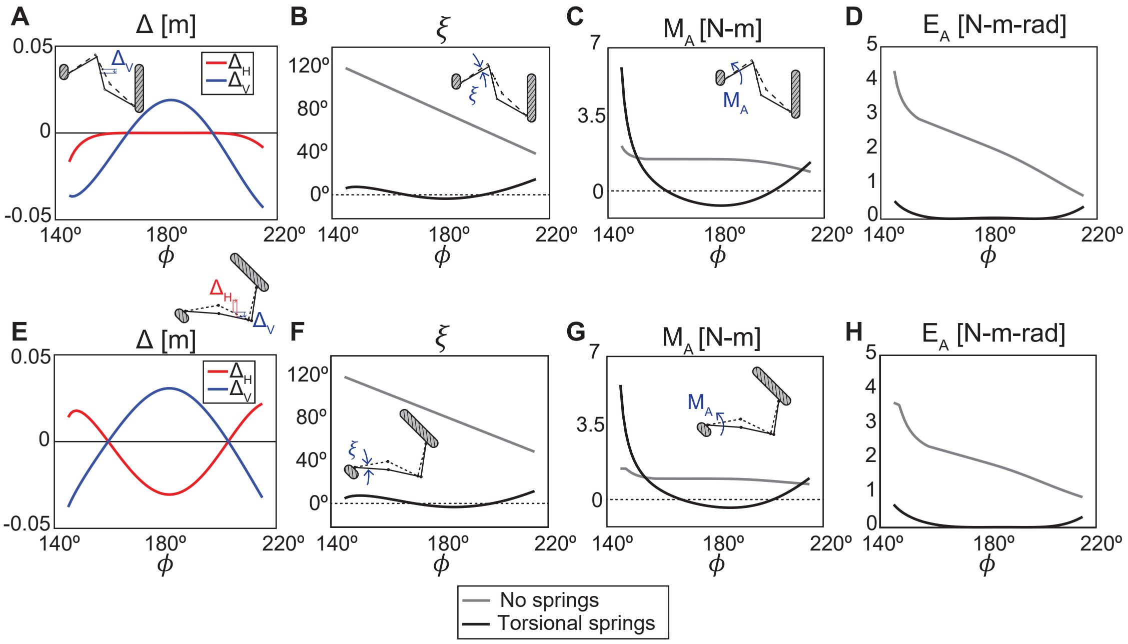

Figure S12: (A) Residual displacements for the Watt's linkage at $\psi = 0°$. (B) Rotation angle ξ between the desired configuration and the equilibrium state. (C) Actuation moment required to move the structure from the equilibrium state to the desired state (i.e., moment required to rotate ξ radians.) (D) Energy required for actuation. (E-H) Similar trends are observed for the Watt's linkage at $\psi = 45°$.

ment, and actuation energy for the Watt's linkage at $\psi = 45°$ (Figure S12(E-H)). Similar trends are seen for all quantities; again, the actuation energy is nearly zero with the addition of torsional springs.

Locking

Locking mechanisms can be used to make a reconfigurable linkage more suitable for load-bearing applications. Fixing all of the rotational DOFs of the Watt's linkage results in an increase in stiffness in both the horizontal and vertical directions (Figure S13(A) and (B)). When all rotational DOFs are locked, the horizontal stiffness of the Watt's linkage depends on both the member cross-sectional area (A) and the moment of inertia (I) (Figure S13(C)). This is because when the structure is locked, the load results in axial and bending deformations. The vertical stiffness, however, only depends on the moment of inertia when the Watt's linkage is fully locked (Figure S13(D)). The structure experiences bending as a result of the vertical load and there is no axial dependence.

The most effective combination of rotational DOFs to lock varies along the kinematic path. For all configurations, locking all rotational DOFs (at locations A, B, C, and D) leads to a system with the highest stiffness in the vertical direction. For horizontal stiffness, however, locking at location combinations BC, ABC, or BCD lead to the same stiffness as combination ABCD for all configurations (Figure S14). At the orientation $\psi = 45°$,

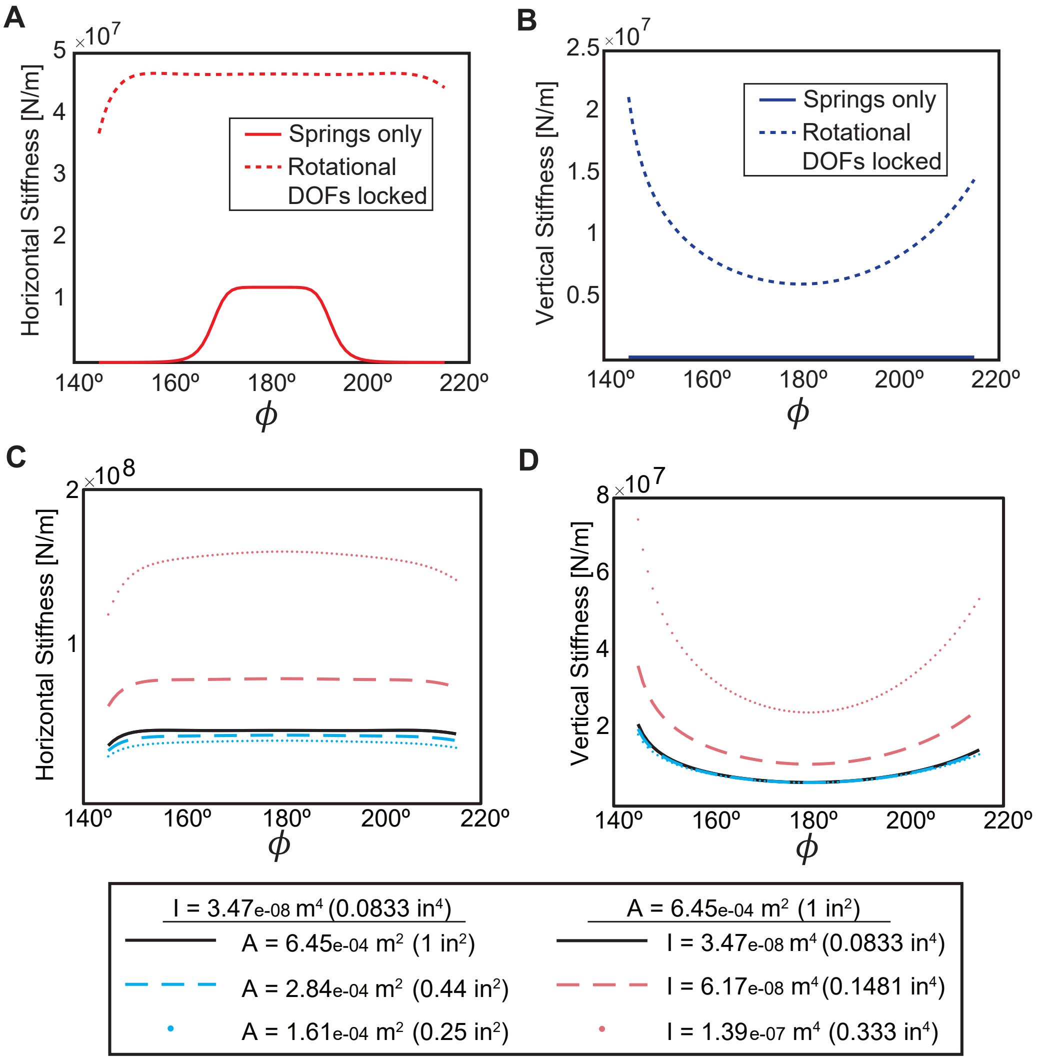

Figure S13: (A) The stiffness of the Watt's linkage in the horizontal direction is increased when all rotational DOFs are locked. (B) In the vertical direction, locking increases the stiffness by several orders of magnitude. (C) The horizontal stiffness of the Watt's linkage depends on the member cross-sectional area (A) and the moment of inertia (I) when all rotational DOFs are locked. (D) The vertical stiffness of the Watt's linkage depends on the moment of inertia and does not depend on the cross-sectional area when all rotational DOFs are locked.

the highest stiffness in either direction is only attainable by locking all four locations, and overall the two directions have stiffnesses along the same order of magnitude (Figure S15. At $\psi = 90°$, locking at location combinations BC, ABC, or BCD predictably all provide the same vertical stiffness as combination ABCD (Figure S15), since the vertical stiffness of the linkage at $\psi = 90°$ is equivalent to its horizontal stiffness at $\psi = 0°$.

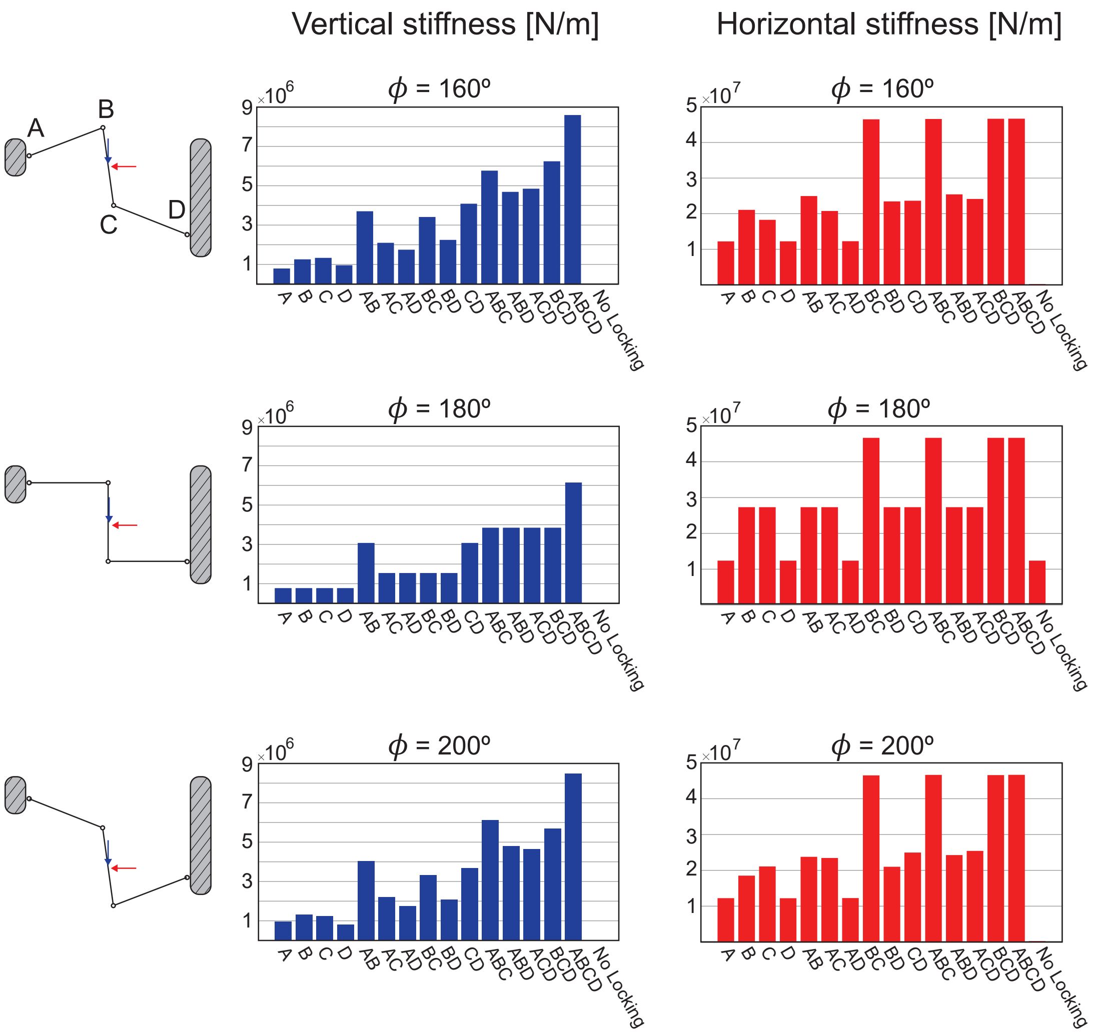

Figure S14: Stiffness of the Watt's linkage at $\psi = 0°$ for different locking combinations. The stiffness of the Watt's linkage can be increased by locking one or more rotational DOFs, at locations A, B, C, and D. The stiffness also changes as the structure reconfigures along its kinematic path. The highest vertical stiffness can be obtained by locking rotations at all locations (ABCD). The largest horizontal stiffness, however, can be achieved by locking location combinations BC, ABC, ACD, or ABCD.

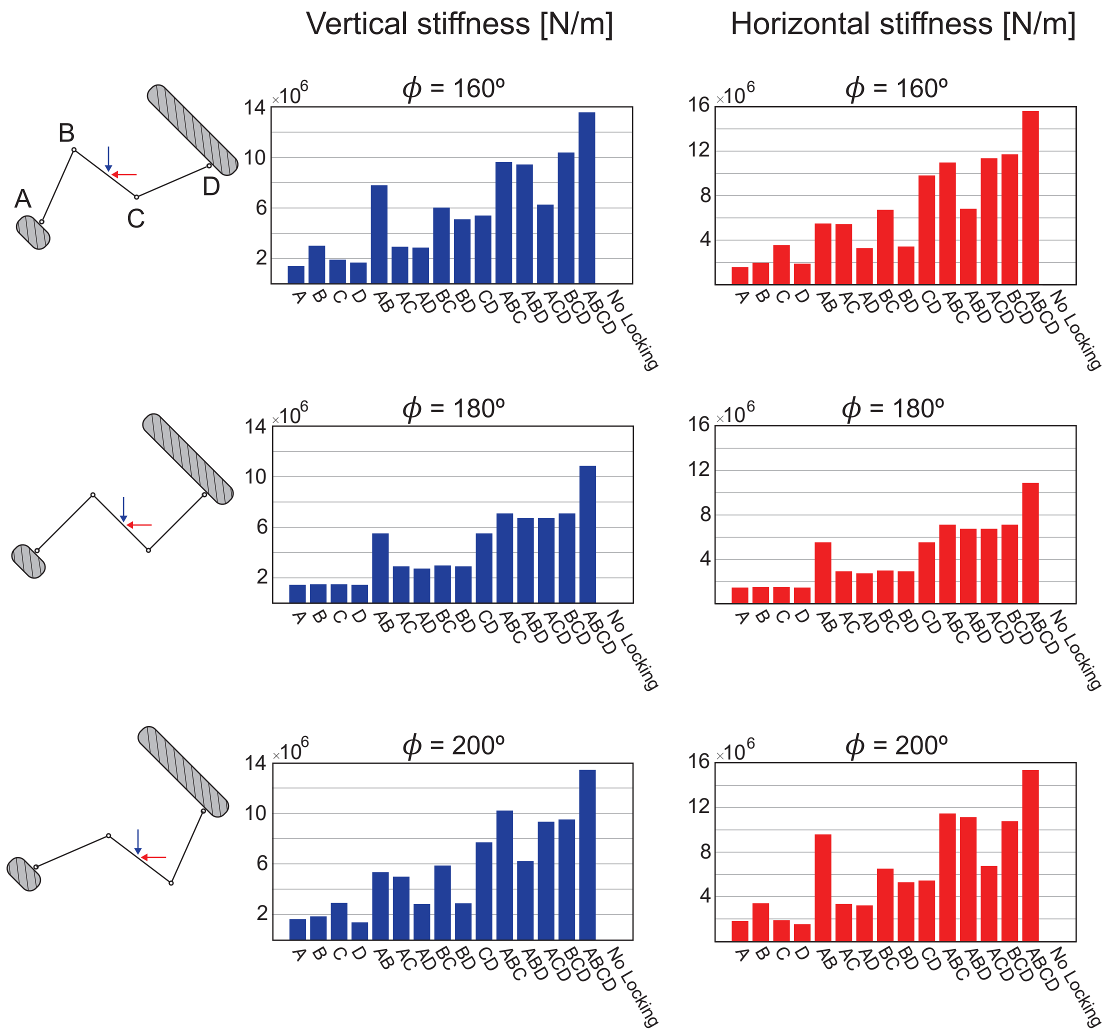

Figure S15: Stiffness of the Watt's linkage at $\psi = 45°$ for different locking combinations. The stiffness of the Watt's linkage can be increased by locking one or more rotational DOFs, at locations A, B, C, and D. The stiffness also changes as the structure reconfigures along its kinematic path. For this orientation, locking more DOFs always increases the stiffness, and the vertical and horizontal stiffness are of the same order of magnitude.

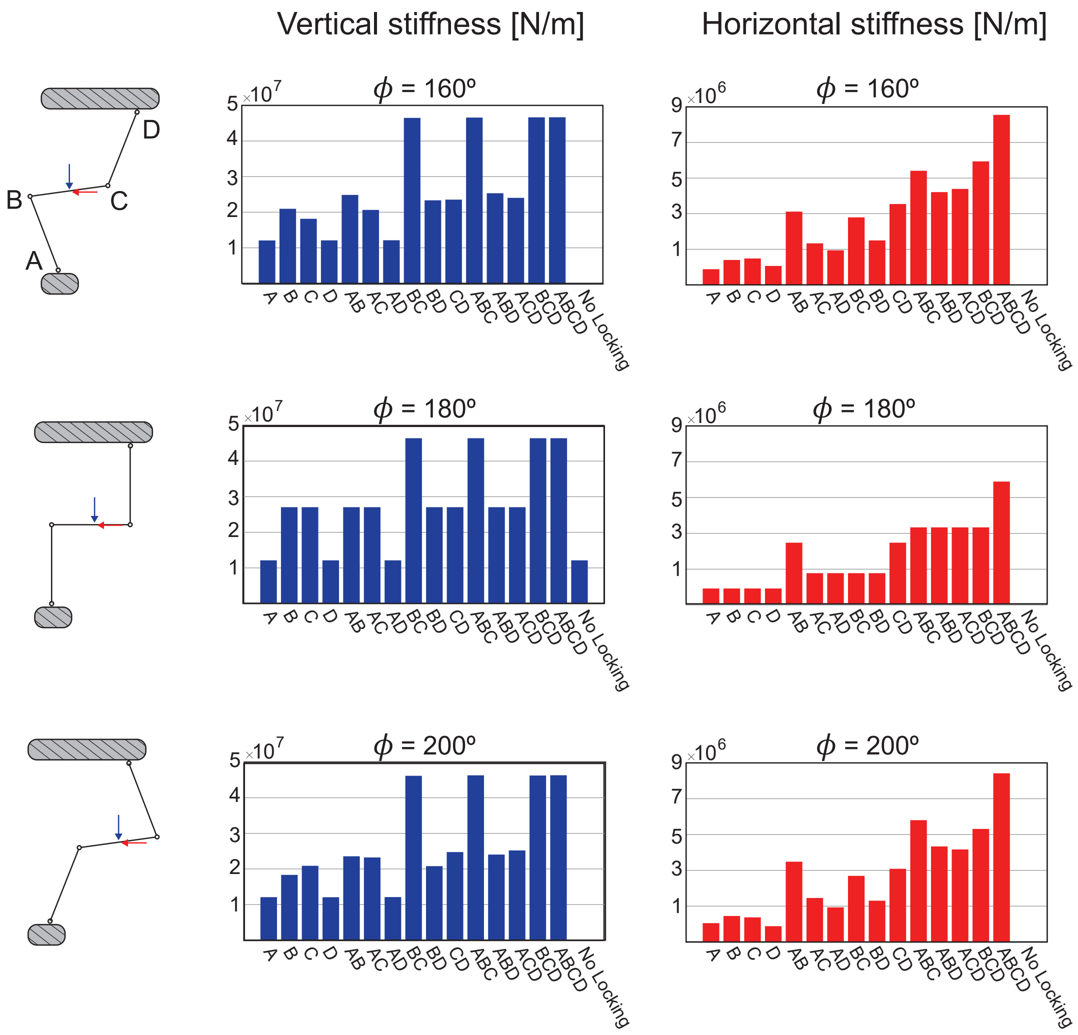

Figure S16: Stiffness of the Watt's linkage at $\psi = 90°$ for different locking combinations. The stiffness of the Watt's linkage can be increased by locking one or more rotational DOFs, at locations A, B, C, and D. The stiffness also changes as the structure reconfigures along its kinematic path. The highest horizontal stiffness can be obtained by locking rotations at all locations (ABCD). The largest vertical stiffness, however, can be achieved by locking location combinations BC, ABC, ACD, or ABCD.

Supplementary files: Movies S1 - S8

Movie S1. Watt's linkage prototype at $\psi = 0°$ with no springs. This system requires a constant force to avoid collapse due to gravity.

Movie S2. Watt's linkage prototype at $\psi = 0°$ with internal torsional springs. This continuous equilibrium system remains stable at all configurations.

Movie S3. Watt's linkage prototype at $\psi = 45°$ with no springs. This system requires a constant force to avoid collapse due to gravity.

Movie S4. Watt's linkage prototype at $\psi = 45°$ with internal and external torsional springs. This system has been reoriented and maintains continuous equilibrium, and remains stable at all configurations.

Movie S5. Watt's linkage prototype at $\psi = 90°$ with no springs. This system is in continuous equilibrium in a small range in the center of the kinematic path, but collapses when moved outside of this range.

Movie S6. Watt's linkage prototype at $\psi = 90°$ with internal and external torsional springs. This system has been reoriented and maintains continuous equilibrium, and remains stable at all configurations.

Movie S7. Scissor Mechanism at $\psi = 0°$ with no springs.

Movie S8. Scissor Mechanism at $\psi = 0°$ with internal torsional springs.
